# Utilization Barriers and Medical Outcomes Commensurate With the Use of Telehealth Among Older Adults: Systematic Review

**DOI:** 10.2196/20359

**Published:** 2020-08-12

**Authors:** Clemens Kruse, Joanna Fohn, Nakia Wilson, Evangelina Nunez Patlan, Stephanie Zipp, Michael Mileski

**Affiliations:** 1 School of Health Administration Texas State University San Marcos, TX United States

**Keywords:** telehealth, telemedicine, older adults, barriers, health outcomes

## Abstract

**Background:**

Rising telehealth capabilities and improving access to older adults can aid in improving health outcomes and quality of life indicators. Telehealth is not being used ubiquitously at present.

**Objective:**

This review aimed to identify the barriers that prevent ubiquitous use of telehealth and the ways in which telehealth improves health outcomes and quality of life indicators for older adults.

**Methods:**

This systematic review was conducted and reported in accordance with the Kruse protocol and the Preferred Reporting Items for Systematic Reviews and Meta-Analyses (PRISMA) guidelines. Reviewers queried the following four research databases: Cumulative Index of Nursing and Allied Health Literature (CINAHL), PubMed (MEDLINE), Web of Science, and Embase (Science Direct). Reviewers analyzed 57 articles, performed a narrative analysis to identify themes, and identified barriers and reports of health outcomes and quality of life indicators found in the literature.

**Results:**

Reviewers analyzed 57 studies across the following five interventions of telehealth: eHealth, mobile health (mHealth), telemonitoring, telecare (phone), and telehealth video calls, with a Cohen κ of 0.75. Reviewers identified 14 themes for barriers. The most common of which were technical literacy (25/144 occurrences, 17%), lack of desire (19/144 occurrences, 13%), and cost (11/144 occurrences, 8%). Reviewers identified 13 medical outcomes associated with telehealth interventions. The most common of which were decrease in psychological stress (21/118 occurrences, 18%), increase in autonomy (18/118 occurrences, 15%), and increase in cognitive ability (11/118 occurrences, 9%). Some articles did not report medical outcomes (18/57, 32%) and some did not report barriers (19/57, 33%).

**Conclusions:**

The literature suggests that the elimination of barriers could increase the prevalence of telehealth use by older adults. By increasing use of telehealth, proximity to care is no longer an issue for access, and thereby care can reach populations with chronic conditions and mobility restrictions. Future research should be conducted on methods for personalizing telehealth in older adults before implementation.

**Trial Registration:**

PROSPERO CRD42020182162; https://www.crd.york.ac.uk/prospero/display_record.php?ID=CRD42020182162.

**International Registered Report Identifier (IRRID):**

RR2-10.2196/15490

## Introduction

### Background

A demographic shift has been evident globally since 2015. Specifically, the aging population has been growing at a rapid rate and has been predicted to reach 22% by the year 2050 [1]. In fact, the World Health Organization (WHO) estimates that during 2020, adults aged 60 years or older will outnumber children aged 5 years or younger [1]. The United States Census Bureau published a graphic on March 13, 2018, depicting the population pyramid from 1960 and comparing it with the 2060 prediction [2]. The graphic demonstrated the gradual change of the US population pyramid to a pillar shape [2]. This graphic is key to understanding the demands on the health care system in the area of geriatric, long-term, and end-of-life care, because it highlights the larger number of older adults living longer lives. By 2030, 60 million people in the “baby boomer” generation (born between 1946 and 1964) will have reached 65 years of age or older and will be eligible for age-related state entitlements in most countries [3,4]. This demographic shift is an impending issue facing health care, as geriatric, long-term, and end-of-life care will experience a surge in demand. Health care organizations and their providers must find ways to effectively allocate resources and provide the right care at the right time and at the right place [5].

Telemedicine has the potential to increase access among elderly people and relieve the stress regarding care for the unusually large number of elderly people. The WHO defines telemedicine as “healing from a distance.” More specifically, it is healing through the use of information and communication technologies “to improve patient outcomes by increasing access to care and medical information” [6]. The WHO also does not differentiate between the terms telemedicine and telehealth.

There has not been much work on the use of telehealth based on age; however, we know that a technology gap or digital divide exists. It is established by tiers of race, age, and economic disparities [7]. In the United States, for instance, the elder-care entitlement Medicare imposes restrictions on the use of telehealth for the primary population [8]. The Coronavirus Aid, Relieve, and Economic (CARES) Act provides a regulatory waiver to extend reimbursements to telemedicine, but this is only a relief act and not permanent legislation [9]. Previous reviews have investigated facilitators and barriers to the adoption of telehealth, the use of eHealth and mobile health (mHealth) tools in health promotion and primary prevention among older adults, and patient satisfaction with telehealth interventions [10-12]. A narrative analysis on mHealth solutions for the aging population used a generational analysis that included culture and trust of other people and a distrust of technology [13]. This work noted an increase in the use of technology for health purposes and an increase in the use of the internet for health purposes. It also noted concerns of security and privacy and technical troubleshooting. A review from 6 years ago spanned 10 years, analyzed 14 articles, and focused on older adults over 65 years old [10]. The most recent review on a topic most like this work was published 5 years ago, spanned 10 years, analyzed 45 articles, and focused on older adults aged over 50 years [11].

With an aging population, telehealth services are becoming more common to aid in independent living and health management [14]. An example of telehealth is virtual home health care, where health care providers provide guidance in specific procedures while the patients are in the comfort of their home. Telehealth programs can improve access to health care and have a positive effect on patients’ medical outcomes, especially for the treatment of chronic illnesses in vulnerable populations, such as elderly people [15]. Utilizing age-friendly technology could improve the care providers give to older adults through telehealth services and improve the usability of telehealth for older adults [16]. It is essential to first understand the barriers that affect the usability of telehealth services among older adults in order to find opportunities for improving health outcomes. Barriers to using telehealth can affect the accessibility of health services to older adults. When it comes to technology, older adults are often stereotyped as laggards in technology adoption [7]. However, owing to rising telehealth capabilities, improvement of access, especially to older adults, can aid in improving health outcomes [15]. Understanding the perspectives of older adults is important when evaluating telehealth barriers because older adults generally develop different perspectives compared with those of other age demographics [16]. Other studies on this topic have focused on conditions like depression, heart failure, and falls [17-19]. However, no review has looked at medical outcomes, including indicators of quality of life, that come as a benefit of using telehealth and the barriers that exist to the use of telehealth internationally. This review intends to examine these issues and what has changed in telehealth for older adults in the last 5 years.

### Objectives

The purpose of this systematic review was to evaluate the current literature to help identify and understand health-related quality of life enhancers and general health outcomes that are commensurate with and barriers to the use of telehealth services by older adults. Health outcomes, including quality of life enhancers, provide the “so what” to the use of telehealth modalities. Recognizing barriers can help develop solutions for broadening the use of telehealth services in older adults. During the COVID-19 crisis, providers and patients alike were thrust into the world of telehealth. An overview of the benefits and barriers would be helpful to those deciding whether to continue the use of telehealth modalities.

## Methods

### Protocol and Registration

This review used the Kruse protocol published in 2019 and the Preferred Reporting Items for Systematic Reviews and Meta-Analyses (PRISMA) guidelines [20,21]. The review was registered with PROSPERO on May 2, 2020 (ID: CRD42020182162). In accordance with the rules at PROSPERO, the registration was completed before analysis began.

### Eligibility Criteria

Studies were eligible for this review if participants were older adults (older than 50 years), if the intervention was some form of telehealth (including mHealth, eHealth, and all forms of telehealth), if the authors reported either barriers to the use of telehealth or health outcomes, and if the article was published in a research journal in the English language in the last 5 years. Adults older than 50 years were chosen out of trial and error. When we initially wrote the methods for this study, we chose a more universal definition of older adults as those over 65 years of age. Once we started filtering articles for analysis, we noticed a large number of articles that were being eliminated, despite the high level of quality of these studies. If we had stuck with age over 65 years as our screening criteria, we would have eliminated more than half of the group of articles for analysis. As a result, we chose age over 50 years, which is supported by other reviews in this field [11]. This is a limitation we list later.

### Information Sources

The following four databases were queried: Cumulative Index of Nursing and Allied Health Literature (CINAHL), PubMed (MEDLINE), Web of Science (WoS), and Embase (Science Direct). Additionally, a specific journal search was conducted in the journal of choice for publication (Journal of Medical Internet Research). Databases were filtered for the last 5 years. Database searches occurred between February 2 and 14, 2020. A period of 5 years was chosen because it has been that long since the last review was published on a similar topic. We expect to find advances in technology and advances in adoption by elderly people because younger people who use technology regularly have advanced into the observation group of over 50 years old. We hope to find fewer barriers.

### Search

Reviewers carefully analyzed the MEDLINE Medical Subject Headings (MeSH) for key terms related to telehealth and elderly people. Based on the established hierarchy of indexed terms at MeSH and a series of experimental searches, the final search terms were “Telehealth AND ‘older adults.’” This combination of terms yielded the maximum number of results in all four databases. Reviewers used available filters to eliminate other reviews and focus on academic or peer-reviewed journals over the last 5 years.

### Study Selection

Reviewers followed the Kruse protocol, which entails a series of three consensus meetings. The results of the first consensus meeting identified the studies for analysis. After filtering the results of the four databases to meet the eligibility criteria, all reviewers screened the abstracts of the results to ensure that articles were germane to the topic, they were actually studies (not protocols), and they contained tangible results to enable analysis toward the review’s objectives. The first consensus meeting discussed whether to keep articles for analysis. The reasons for rejection included opinion article (not a study), protocol (no results), concept or design paper (no results), review, no use of telehealth, and no reporting of either outcomes or barriers. A kappa statistic was calculated from the results of this meeting [20]. Before consensus meeting number two, the group leader assigned workload to ensure that each article was analyzed by at least two reviewers. Reviewers independently analyzed articles using a piloted form. Reviewers collected several standard items used for summary, such as PICOS (Participants, Intervention, Comparison [to the control group], Outcome, Study design), and analysis, such as forms of telehealth interventions, barriers to the use of telehealth by older adults, and the medical outcomes observed in older adults using telehealth solutions [20]. After making a list of observations, reviewers attempted to make sense of the observations using a narrative analysis [22].

### Data Collection Process

The group leader divided analysis workload to ensure all articles were reviewed by at least two reviewers. Reviewers independently analyzed articles using a standardized Excel spreadsheet as a piloted form for data extraction.

### Data Items

The piloted form collected data, including participants, intervention, study design, results compared to a control group (where applicable), medical outcomes, sample size, bias within studies, effect size, country of origin, statistics used, barriers to the use of telehealth, and quality assessment from the John Hopkins Nursing Evidence-Based Practice (JHNEBP) rating scale, as well as general observations about the article that would help in interpretation of the results [23]. These data items were independently collected and discussed in the second consensus meeting.

### Risk of Bias Within and Across Studies

General observations of bias were made about each study, such as selection bias. These observations were independently collected and discussed in the second consensus meeting. The JHNEBP rating scale was used to assess the risk and quality of each study analyzed. Within the JHNEBP rating scale, level I indicates experimental studies, randomized controlled trials (RCTs), or meta analyses of RCTs; level II indicates quasiexperimental studies; level III indicates nonexperimental studies, qualitative studies, or meta-syntheses; level IV indicates opinions of nationally recognized experts based on research evidence or expert consensus panels (systematic reviews or clinical practice guidelines); and level V indicates opinions of individual experts based on nonresearch evidence. There are three levels of quality of evidence, which are listed as A (high quality), B (good quality), and C (low quality or major flaws). Each of these levels define the following four thresholds: research, summative reviews, organizational opinion, and expert opinion. For instance, in level A, studies have consistent results with sufficient sample size, adequate control, and definitive conclusions. In level C, studies have little evidence with inconsistent results and insufficient sample size, and conclusions cannot be drawn. To limit the inherent bias and limitations commensurate with low-quality studies, the ratings from the JHNEBP rating scale serve as screening criteria. Articles with evidence ratings below level IV were not accepted. Quality of evidence ratings below level B were highly suspect.

### Summary Measures and Additional Analysis

The review analyzed both qualitative and quantitative methods, so the summary measures sought were not consistent. The preferred summary statistic was the risk ratio, but other summary statistics were also sufficient. The summary statistics were independently collected and discussed in the second consensus meeting.

A narrative analysis summarized themes for barriers, interventions, and medical outcomes. They were reported in summary statistics in affinity matrices. These themes were independently collected and discussed in the third consensus meeting. After themes were identified, interactions between themes were observed using a spreadsheet.

## Results

### Study Selection

**Figure 1:**
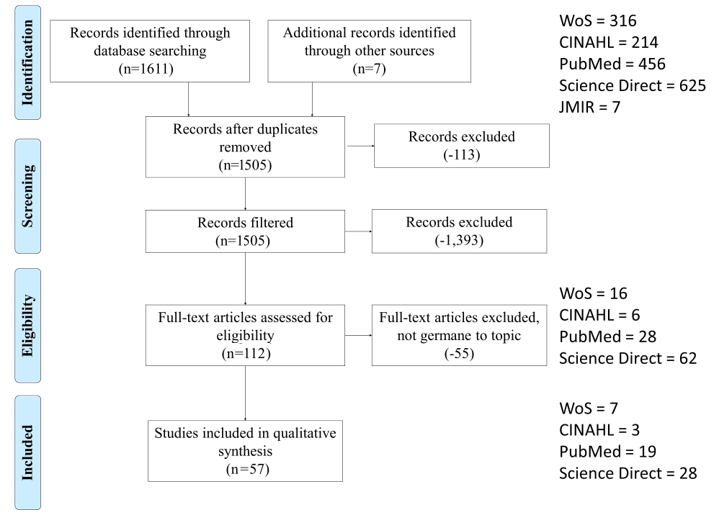
Study selection process.

Figure 1 illustrates the study selection process. A kappa statistic was calculated to measure the reliability of article selection between reviewers. The κ value was 0.75, representing moderate agreement [24,25].

### Study Characteristics

Table 1 lists the ancillary data extracted from the studies analyzed in reverse chronological order as follows: 2020 [26], 2019 [5,26-34], 2018 [4,15,16,35-46], 2017 [14,47-56], 2016 [19,57-63], and 2015 [64-76].

**Table 1:** PICOS characteristics.

Authors, year	Participants	Intervention	Comparator	Medical outcomes reported	Study design
Hamilton et al, 2020 [26]	765 older adults; ≥55 years; Medicare/Medicaid beneficiaries; English 76% (581), Spanish 20% (153), and others 4% (31); low income	Telemonitoring Remote patient monitoring (RPM): blood-pressure cuffs, pulse oximeters, and body weight scales Telehealth Intervention Programs for Seniors, RPM, extensive social wraparound services, care coordination, and intergenerational socialization aimed at improving health care options to assist low-income high health risk older adults who live in subsidized congregate housing or attend local community centers for older adults. A survey instrument was collected each week.	None	Hospital visits and readmissions	Observational study
Theis et al, 2019 [5]	551 older adults, ≥60 years, 51.3% male and 48.7% female 441 participants (80%) already retired, 109 (19.8%) still working	eHealth	None	Satisfaction: 64% (353) of older adults were satisfied with the health information they received, 34% (187) were neutral, and 2% (11) were dissatisfied	Analytical observational study
Wildenbos et al, 2019 [27]	13 older adults, ≥50 years, primarily Dutch speaking Additional inclusion criteria for App 2: heart failure (HF) patient and chronic obstructive pulmonary disease	mHealth^a^ Investigated these interaction issues in two different case studies; an app for older adults facilitating their hospital appointment attendance (App 1) and a self-monitoring app for chronically ill older patients	None	Cognitive impairment was reported but not compared with a control.	Case study
Jakobsson et al, 2019 [28]	9 older adults, 65-85 years, cognitive impairment of different origin (eg, stroke, dementia, and mild cognitive impairment)	Telehealth, smartphone, computer, and landline	None	Cognitive impairment was reported but not compared with a control.	Qualitative study
Karlsen et al, 2019 [29]	18 older adults, ≥60 years, living in their own homes and having recently received telecare service (within the last 0-3 months), received home care services, Norwegian speaking, no limitations considering disease or chronic conditions	Telemonitoring Personal alarm (16), light sensors (3), stove alarm (4), GPS tracking (3), medication reminders (8), bed sensors (1), door sensor (2), video surveillance (2)	None	Safety, satisfaction, security, independence, responsibility, mindfulness of failty	Qualitative study
Coley et al, 2019 [30]	341 (quantitative) and 46 (qualitative) older adults; ≥65 years; Finland, France, and Netherlands; response rate 79% (Finland: 81%, France: 72%, Netherlands: 87%, *P*=.04); 48% (164) male; 51% (174) university-level education	eHealth Participants were randomized to either an interactive internet platform designed to encourage goal setting and lifestyle changes with the remote support of a lifestyle coach or a control platform with basic health information but no interactive features or coach support. Owing to the nature of the intervention, complete double blinding was not possible, but masking was attempted by informing participants that they would be randomized to one of two internet platforms (without further details on the content).	Control	Not reported	Cross-sectional mixed-methods randomized controlled trial (RCT)
Giesbrecht & Miller, 2019 [31]	18 older adults, ≥50 years, resided in the community, self-propelled using both hands at least 1 hour per day inside and outside their home, English speaking	eHealth The treatment group incorporated two in-person training sessions with a trainer and 4 weeks of monitored home training using a computer tablet (mHealth) wheelchair skills program. The control group did not receive skills training, as is typical practice with this population.	Control	Skill capacity and safety	RCT
Brodbeck et al, 2019 [32]	110 older adults, >50 years, 79% (87) female	eHealth Internet-based self-help intervention for prolonged grief symptoms after spousal bereavement or separation/divorce	Control	Grief, depression, psychological distress, embitterment, loneliness, and life satisfaction	Mixed methods, RCT
Mosley et al, 2019 [33]	112 older adults, ≥60 years, 58% (65) female, English speaking	eHealth Etymotic home hearing test compared with traditional manual audiometry	Control	Not reported	Quasiexperimental study
Jensen et al, 2019 [34]	20 older adults, hip fracture	eHealth “My Hip Fracture Journey” on iPad (provided) education through pictographs, video clips, illustrated exercises, and written information. This was used to augment home visits and subsequent interviews.	None	Autonomy and self-care	Qualitative study
Rasche et al, 2018 [15]	576 older adults, ≥60 years, 48.7% (280) female, German speaking	eHealth The national survey queried the use of health apps and their perceived usefulness.	Control	Not reported	Quasiexperimental study
Portz et al, 2018 [35]	30 older adults, ≥60 years, location at the University of Colorado Hospital and the University Hospital Cleveland Medical Center in Cleveland (Ohio), 60% (18) female, 63% (19) black people	mHealth The HF app was developed to allow patients to track their symptoms of HF. Thirty older adults completed an acceptability survey after using the mobile app. The survey used Likert items and open-ended feedback questions.	None	Awareness of the condition and self-care	Quantitative acceptability survey analysis
Castro et al, 2018 [36]	501 older adults, ≥65 years, Medicare population	eHealth Participants were matched into geographically based small groups with an assigned health coach, and they began the program at the same time. Group members were connected to each other through a private online social forum where they could post comments and questions, engage in health coach–moderated discussions, and provide social support to one another. Using internet-enabled devices (laptop, tablet, or smartphone), program participants were able to asynchronously complete weekly interactive curriculum lessons, reflections, and goal-setting activities in relation to the weekly topic.	Pretest	Weight: participants lost an average of 13-14 pounds (8%) HbA_1c_: 0.14% absolute decrease at 6 months and 12 months (*P*<.001) Cholesterol: mean reduction of -12.92 mg/dL (*P*<.001).	Single-arm pretest/posttest design
Joe et al, 2018 [37]	43 older adults, 70% (30) female	eHealth A focus-group method was used to brainstorm designs for telehealth for older adults.	None	Not reported	Qualitative analysis study (focus groups)
Dham et al, 2018 [4]	134 older adults, 60% (80) female	Telemedicine Telepsychiatry assessments	None	Not reported	Mixed-methods cross-sectional cohort study with retrospective chart review and prospective feedback survey
Paige et al, 2018 [16]	384 older adults, 74.3% (285) female, 57.7% (222) Caucasian people, 42.3% (162) black people	eHealth eHealth awareness and eHealth literacy scale	None	Not reported	Qualitative measurement invariance study
Cajita et al, 2018 [38]	10 older adults, ≥65 years, history of HF, spoke English, difficulty with mobile technology	mHealth	None	Not reported	Descriptive exploratory study
Harte et al, 2018 [39]	22 older adults, >65 years, difficulty using smartphones	mHealth Training on a smartphone-based fall detection and prevention system	None	Not reported	Usability and learnability case study
Gordon & Hornbrook, 2018 [40]	2602 older adults, >65 years, 54% (1,405) female, 79% (2,056) Caucasian people	eHealth Online forms, online tracking systems, and patient portal	None	Not reported	Mixed-methods cross-sectional study
Bao et al, 2018 [41]	12 older adults, ≥65 years, 75% (8) female	eHealth Online training	Pretest	Sensory organization test, mini balance evaluation system test, five times sit to stand test, and no statistical significance in other clinical outcomes	Pretest posttest true experiment
Egede et al, 2018 [42]	241 older adults, >63 years, 98% (236) male, 60% (144) Caucasian people, veterans having major depressive disorder	Telemedicine Telepsychotherapy	Control	Baseline depression severity, generalized anxiety disorder, alcohol misuse, cannabis misuse, and cannabis dependence	RCT
Platts-Mills et al, 2018 [43]	75 older adults, <50 years, musculoskeletal pain	Telecare Telephone call and protocol-guided follow up	Control	Pain	Randomized controlled pilot study
Lopez-Villegas et al, 2018 [44]	50 older adults, >65 years, 48% (24) women, seen in the cardiology clinic, using a pacemaker	Telemonitoring Pacemakers	Control	EQ-5D VAS^b^ (health-related quality of life)	RCT
Dugas et al, 2018 [45]	27 older adults, >60 years	mHealth DiaSocial for glucose control, exercise, nutrition, and medication adherence	None	Glucose management and HbA_1c_	Pilot study
Nalder et al, 2018 [46]	8 older adults, >55 years, type 2 diabetes	eHealth Three internet-based platforms: 1. Chronic disease management 2. Real-world strategy training 3. Learning the ropes	None	HbA_1c_, independence, emotional support, and motivation to self-manage	Qualitative pilot program
Buck et al, 2017 [47]	12 older adults, >60 years, 42% (5) female	eHealth PSHA, a web-based tablet-delivered intervention developed internally, which encourages the participant to record daily medication intake, weight, and time spent with a brief exercise program using an aerobic stepper. The tablet records daily information, and the participant watches a short heart health educational video.	None	Documentation for nutrition and eating and instructional video exposure	Proof-of-concept trial Qualitative semistructured interviews after the study protocol
Ware et al, 2017 [14]	15 older adults, ≥50 years, 73% (11) female	eHealth	None	Not reported	Two focus groups and pragmatic thematic analysis
Chang et al, 2017 [48]	18 older adults, >65 years, diabetes	Telehealth Diabetes management	None	Self-management and independence	Qualitative research design and 1-1 semistructured interviews
Cajita et al, 2017 [49]	129 older adults, >65 years, 73.6% (95) male, 56.6% (73) Caucasian people	mHealth Simple linear regression was used to test the relationship between the main study variables (eHealth literacy, social influence, perceived financial cost, perceived ease of use, and perceived usefulness) and intention to use mHealth.	None	Not reported	Cross-sectional correlational study
LaMonica et al, 2017 [50]	221 older adults, ≥50 years, 57.7% (128) female	eHealth Memory aids and mental acuity exercises	None	Memory	Qualitative study
Bahar-Fuchs et al, 2017 [51]	45 older adults; >65 years; mild cognitive impairment (n=9), mood-related neuropsychiatric symptoms (n=11), or both (n=25)	eHealth Tailored and adaptive computer cognitive training in older adults at risk for dementia	Control	Memory, global cognition, learning, and mood	RCT
Nahm et al, 2017 [52]	866 older adults, >50 years, bone health issues	eHealth Bone Power program	Control	Osteoporosis knowledge, self-efficacy/outcome expectations, and exercise behaviors	Two-arm RCT
Knaevelsrud et al, 2017 [53]	47 older adults, >50 years, 64.9% (31) female, posttraumatic stress disorder symptoms, German speaking	eHealth Internet-based therapist-guided intervention	Control	Comfort (from not being able to see the therapist), satisfaction, motivation, feeling of being understood	RCT
Reijnders et al, 2017 [54]	376 older adults, >50 years, 67.5% female	eHealth Cognitive functioning	Control	Feelings of stability, memory functioning, and locus of control	RCT
Hamblin et al, 2017 [56]	60 older adults, >85 years	Telemonitoring	None	Autonomy, awareness of danger areas like gardens or staircases, and safety	Mixed-methods study
Mageroski et al, 2016 [55]	25 older adults, >50 years	Telemonitoring Remote sensors in homes of older adults	None	Not reported	Mixed-methods study
Wang et al, 2016 [57]	29 older adults, >65 years, 71% (21) female	Telemonitoring Wearables, mobile devices, trackers, and in-home telemonitoring	None	Not reported	Cross-sectional study
Gordon & Hornbrook, 2016 [58]	231,082 older adults for database arm, 2602 older adults for survey arm	eHealth	None	Not reported	Mixed methods, database, and survey study
Williams et al, 2016 [59]	7 older adults, >60 years, dementia	eHealth	None	Not reported	Pilot study
Evans et al, 2016 [19]	41 older adults, >55 years, 57.1% (23) female, English speaking	mHealth Remote monitoring, wrist wearable, and wireless tablet	Control	Documentation for weight and blood pressure	Single-arm quasiexperimental study
Muller et al, 2016 [60]	43 older adults, ≥55 years, mobile phone use, no regular exercise	mHealth SMS and Physical Activity for Health Study	Control	Exercise, mood, fitness, health, mindfulness of the importance of exercise, and guilt	Two-arm parallel RCT
Quinn et al, 2016 [61]	118 older adults, >50 years, 66% (78) female, diabetes	mHealth Mobile diabetes intervention study	Control	HbA_1c_	True experiment
Royackers et al, 2016 [62]	8 older adults, caring for loved ones in their last days	eHealth Point of care technology through eShift (home-based palliative care)	None	Comfort, independence, and autonomy	Qualitative pilot study
Duh et al, 2016 [63]	45 older adults, >60 years	Telecare CareMe	None	Not reported	Qualitative participatory design
Depatie & Bigbee, 2015 [64]	30 older adults, ≥60 years, 80% (24) female	mHealth Mobile health technology for older adults in rural communities	None	Not reported	Mixed-methods study
Moore et al, 2015 [65]	26 older adults, >55 years, 77% (20) male	eHealth Internet-based hearing health care for older adults	None	Not reported	Training study
Currie et al, 2015 [66]	168 older adults, ≥60 years, living in rural areas, long-term chronic pain	eHealth	None	Pain	Mixed-methods study
Grant et al, 2015 [67]	762 older adults, >60 years, 67% (511) female, 90% (686) Caucasian people	Telemonitoring LivingWell@Home, sensors (motion, bed, and humidity), emergency response systems, and biometric monitors (heart rate, blood pressure, weight, pulse oximetry, and blood glucose)	Control	Satisfaction, autonomy, and independence	RCT
Brenes et al, 2015 [68]	141 older adults, ≥60 years, 81% (114) female, living in rural areas, diagnosis of generalized anxiety disorder (GAD)	Telecare Telephone-delivered cognitive behavior therapy and telephone-delivered nondirective supportive therapy	Control	Worry, GAD, depression, and anxiety	RCT
Corbett et al, 2015 [69]	2192 older adults, ≥60 years	eHealth Online cognitive training package	Control	Reasoning, verbal learning, and instrumental activities of daily living	RCT
Mavandadi et al, 2015 [70]	1018 older adults, ≥65 years, 83.2% (847) female, community-dwelling, low-income, mental health symptoms	Telecare SUSTAIN care management system (assessment, monitoring, care management, and brief therapies)	Control	Depressive symptoms, anxiety symptoms, and MH functioning	RCT
Egede et al, 2015 [71]	90 older adults, ≥58 years, 98% (88) male, diagnosis of diabetes	Telemedicine Telepsychotherapy	Control	Geriatric depression scale, Beck depression inventory, and Diagnostic and Statistical Manual, version 4 symptoms	RCT
Chang et al, 2015 [72]	192 older adults, >60 years, 81% (156) female, cardiology diagnoses	Telemonitoring Remote cardiology management	None	Cardiac arrhythmias detected and paroxysmal atrial fibrillation detected	Pilot study
Boulos et al, 2015 [73]	27 older adults, 31 caregivers, 43 healthcare professionals	eHealth LiveWell Parkinson intervention and learning modules	None	Communication of the condition with the provider	Pilot study
Dino & deGuzman, 2015 [74]	82 older adults, demographics not reported	Telemedicine, mHealth, and eHealth	None	Not reported	Structured equation modeling
Czaja et al, 2015 [75]	24 older adults, >60 years, 71% (17) female, 94% (23) Hispanic people, diagnosis of hypertension	Telemonitoring Telehealth system that monitors blood pressure and body weight	Control	Self-management, health, and independence	Randomized feasibility study
Choi et al, 2015 [76]	42 older adults, ≥60 years, 81% (34) female, low-income, homebound, score of 15 or above on the 24-item Hamilton Rating Scale for Depression	Telecare Video tele-problem-solving therapy (PST) to in-person PST and telephone care calls	None	Depressive symptoms, understanding of depression, and social interaction	Qualitative

^a^mHealth: mobile health.

^b^EQ-5D VAS: European health-related quality of life utility with a visual analogue scale.

### Risk of Bias Within Studies

At the study level, reviewers recorded observations of bias. The most frequently observed form of bias was selection bias (asking for volunteers for a research study involving technology will result in volunteers who already gravitate toward technology), which occurred in 7 out of 57 (13%) articles analyzed [15,26,30-32,37,39]. There were six instances of convenience samples from a local population [34,49-52,64]. Both examples of bias limit the external validity of the results.

### Results of Individual Studies

Themes that resulted from the narrative analysis are listed in Table 2. Repetition can be observed in a frame of a theme owing to multiple observations from the same article for that theme. Translations from observations to themes for interventions, medical outcomes, and barriers are listed in Multimedia Appendix 1, Multimedia Appendix 2, and Multimedia Appendix 3, respectively. These appendices illustrate the logical inference reviewers made for each theme. For instance, one article listed remote patient monitoring for blood pressure, pulse oximeter, and body weight scales. These were categorized under telemonitoring [26]. The same article listed a decrease in hospital visits and a decrease in readmissions. These were categorized under an increase in hospital metrics. Additional data collected (bias, statistics, country of origin, and quality assessments) are displayed in Multimedia Appendix 4. In consensus meeting number two, we identified general observations, as depicted in the tables [20].

**Table 2:** Summary of the analysis of each article.

Authors, year	Intervention	Medical outcome theme	Theme of barriers
Hamilton et al, 2020 [26]	Telemonitoring	Increase in hospital metrics	Not reported
Theis et al, 2019 [5]	eHealth	Increase in satisfaction	Medical literacy Trust of the internet Ownership of technology^a^
Wildenbos et al, 2019 [27]	mHealth^b^	Increase in cognitive ability	Visual acuity^a^ Mental acuity Technical literacy
Jakobsson et al, 2019 [28]	mHealth eHealth Telecare (phone)	Increase in cognitive ability	Social implications Privacy and security^a^ Technical literacy Lack of desire Ownership of technology Lack of technical support
Karlsen et al, 2019 [29]	Telemonitoring	Increase in safety or security Increase in health-related quality of life Increase in safety or security^a^ Increase in autonomy Increase in mindfulness of the condition	Mental acuity Visual acuity Social implications
Coley et al, 2019 [30]	eHealth	Not reported	Trust of the internet
Giesbrecht & Miller, 2019 [31]	eHealth	Increase in cognitive ability Increase in safety or security	Not reported
Brodbeck et al, 2019 [32]	eHealth	Decrease in psychological distress^a^ Increase in health-related quality of life	Not reported
Mosley et al, 2019 [33]	eHealth	Not reported	Cost
Jensen et al, 2019 [34]	eHealth	Increase in autonomy^a^	Privacy and security Ownership of technology Lack of desire Lack of technical support Technical literacy
Rasche et al, 2018 [15]	eHealth	Not reported	Trust of the internet Technical literacy^a^ Privacy and security
Portz et al, 2018 [35]	mHealth	Increase in mindfulness of the condition Increase in autonomy	Technical literacy Medical literacy
Castro Sweet et al, 2018 [36]	eHealth	Decrease in medical conditions surrounding diabetes^a^	Not reported
Joe et al, 2018 [37]	eHealth	Not reported	Visual acuity^a^ Hand-eye coordination Technical literacy Lack of desire
Dham et al, 2018 [4]	Telehealth video call	Increase in satisfaction	Visual acuity Auditory acuity
Paige et al, 2018 [16]	eHealth	Not reported	Technical literacy Trust of the internet
Cajita et al, 2018 [38]	mHealth	Not reported	Medical literacy Mental acuity Lack of desire Technical literacy Ownership of technology Cost
Harte et al, 2018 [39]	mHealth	Not reported	Technical literacy
Gordon & Hornbrook, 2018 [40]	eHealth	Not reported	Cost Technical literacy
Bao et al, 2018 [41]	eHealth	Increase in cognitive ability Increase in activity or coordination^a^	Not reported
Egede et al, 2018 [42]	Telehealth video call	Decrease in psychological distress^a^ Decrease in medical conditions surrounding pain^a^	Not reported
Platts-Mills et al, 2018 [43]	Telecare (phone)	Decrease in medical conditions surrounding pain	Not reported
Lopez-Villegas et al, 2018 [44]	Telemonitoring	Increase in health-related quality of life	Not reported
Dugas et al, 2018 [45]	mHealth	Decrease in medical conditions surrounding diabetes^a^	Not reported
Nalder et al, 2018 [46]	eHealth	Decrease in medical conditions surrounding diabetes Increase in autonomy Decrease in psychological distress Increase in autonomy	Technical literacy
Buck et al, 2017 [47]	eHealth	Increase in documentation to give the provider Increase in mindfulness of the condition	Technical literacy
Ware et al, 2017 [14]	eHealth	Not reported	Trust of the internet Medical literacy Technical literacy Social implications Lack of technical support Privacy and security
Chang et al, 2017 [48]	mHealth	Increase in autonomy^a^	Cost
Cajita et al, 2017 [49]	mHealth	Not reported	Medical literacy Lack of desire Cost Technical literacy Social implications
LaMonica et al, 2017 [50]	eHealth	Increase in cognitive ability	Auditory acuity Cost Auditory acuity
Bahar-Fuchs et al, 2017 [51]	eHealth	Increase in cognitive ability^a^ Decrease in psychological distress	Not reported
Nahm et al, 2017 [52]	eHealth	Increase in mindfulness of the condition Increase in autonomy Increase in activity or coordination	Not reported
Knaevelsrud et al, 2017 [53]	eHealth	Increase in safety or security Increase in satisfaction Increase in autonomy Increase in health-related quality of life	Not reported
Reijnders et al, 2017 [54]	eHealth	Increase in activity or coordination Increase in cognitive ability Increase in autonomy	Not reported Privacy and security
Hamblin et al, 2017 [56]	Telemonitoring	Increase in autonomy Increase in mindfulness of the condition Increase in safety or security	Technical literacy Medical literacy Social implications^a^
Mageroski et al, 2016 [55]	Telemonitoring	Not reported	Cost
Wang et al, 2016 [57]	Telemonitoring	Not reported	Lack of desire
Gordon & Hornbrook, 2016 [58]	eHealth	Not reported	Ownership of technology Lack of technical support Cost Technical literacy Hand-eye coordination Trust of the internet Social implications Lack of desire
Williams et al, 2016 [59]	eHealth	Not reported	Technical literacy Lack of technical support Mental acuity Visual acuity Hand-eye coordination
Evans et al, 2016 [19]	mHealth	Increase in documentation to give the provider	Lack of desire Technical literacy Lack of desire Ownership of technology
Muller et al, 2016 [60]	mHealth	Increase in activity or coordination^a^ Decrease in psychological distress Decrease in medical conditions surrounding diabetes Increase in mindfulness of the condition Increase in guilt	Lack of desire
Quinn et al, 2016 [61]	mHealth	Decrease in medical conditions surrounding diabetes	Visual acuity Auditory acuity
Royackers et al, 2016 [62]	eHealth	Increase in safety or security Increase in autonomy^a^	Not reported
Duh et al, 2016 [63]	Telecare (phone)	Not reported	Mental acuity Lack of desire Lack of technical support Technical literacy Cost
Depatie & Bigbee, 2015 [64]	mHealth	Not reported	Cost Lack of desire Social implications Technical literacy Lack of technical support Privacy and security
Moore et al, 2015 [65]	eHealth	Not reported	Technical literacy Computer anxiety Lack of technical support
Currie et al, 2015 [66]	eHealth	Decrease in medical conditions surrounding pain	Social implications
Grant et al, 2015 [67]	Telemonitoring	Increase in health-related quality of life Increase in autonomy^a^	Lack of desire Cost Privacy and security
Brenes et al, 2015 [68]	Telecare (phone)	Decrease in psychological distress^a^	Not reported
Corbett et al, 2015 [69]	eHealth	Increase in cognitive ability^a^ Increase in health-related quality of life	Not reported
Mavandadi et al, 2015 [70]	Telecare (phone)	Decrease in psychological distress^a^	Not reported
Egede et al, 2015 [71]	Telehealth video call	Decrease in psychological distress^a^	Not reported
Chang et al, 2015 [72]	Telemonitoring	Increase in mindfulness of the condition^a^	Not reported
Boulos et al, 2015 [73]	eHealth	Increase in documentation to give the provider	Medical literacy Lack of technical support Mental acuity Hand-eye coordination Visual acuity
Dino & deGuzman, 2015 [74]	mHealth eHealth Telemonitoring	Not reported	Lack of desire Lack of technical support
Czaja et al, 2015 [75]	Telemonitoring	Increase in autonomy^a^ Decrease in medical conditions surrounding diabetes	Technical literacy
Choi et al, 2015 [76]	Telehealth video call	Decrease in psychological distress Increase in mindfulness of the condition Increase in autonomy	Ownership of technology Lack of desire

^a^Multiple uses of this theme in the same article. See Multimedia Appendix 1 for a complete list of individual observations and their translation to themes.

^b^mHealth: mobile health.

### Risk of Bias Across Studies and Quality Assessments

Table 3 summarizes the quality indicators identified by the JHNEBP tool [15]. The most frequent strength rating was III, followed by I, II, and IV. The most frequent evidence rating was A, followed by B and C. No strengths below IV were encountered. A full list of quality assessments is presented in Multimedia Appendix 4. Articles that did not meet the minimum standards of quality were not included in the analysis. This decision was made to limit the bias inherent to nondata-driven opinions or conclusions that do not logically follow the data.

**Table 3:** Summary of quality indicators.

Quality indicator		Value (N=57), n (%)
**Strength of evidence**		
	I (experimental study, RCT^a^, or meta-analysis of RCT)	18 (32%)
	II (quasiexperimental study)	10 (17%)
	III (nonexperimental, qualitative, or meta-synthesis study)	28 (49%)
	IV (opinion)	1 (2%)
**Quality of evidence**		
	A (high quality)	33 (58%)
	B (good quality)	23 (40%)
	C (low quality or major flaws)	1 (2%)

^a^RCT: randomized controlled trial.

### Additional Analysis

The results of consensus meeting three identified the themes that corresponded with telehealth interventions, barriers to the use of telehealth, and medical outcomes. These are summarized in Tables 4-6.

### Interventions of Telehealth

Five themes for interventions were identified. Two studies used multiple telehealth interventions. Table 4 lists the interventions with the associated references, number of occurrences, and probability of occurrence in the review. The most common intervention was eHealth (computer driven), followed by mHealth (smart device driven), telemonitoring (remote sensors), telecare (phone), and telehealth video call.

**Table 4:** Affinity matrix for telehealth interventions.

Intervention	References	Number of occurrences (N=62)	Probability of occurrence
eHealth	[5,14-16,28,30-34,36,37,40,41,46,47,50-54,58,59,62,65,66,69,73,74]	29	47%
mHealth^a^	[19,27,28,35,38,39,45,48,49,60,61,64,74]	13	21%
Telemonitoring	[26,29,44,55-57,67,72,74,75]	10	16%
Telecare (phone)	[28,43,63,68,70]	5	8%
Video call	[4,29,42,71,76]	5	8%

^a^mHealth: mobile health.

### Medical Outcomes and Health-Related Quality of Life Enhancers

Thirteen themes and one observation that did not correspond with a theme for medical outcomes and quality of life factors were reported. Table 5 lists the outcomes with their associated references, number of occurrences, and probability of occurrence in this review. The most common theme for medical outcomes associated with telehealth interventions was *decrease in psychological distress* (decrease in anxiety symptoms, decrease in depressive symptoms, decrease in embitterment, decrease in grief, decrease in worry, decrease in loneliness, increase in emotional support, and increase in mood), with 21 of 118 (18%) occurrences [32,42,46,51,60,68,70,71,76]. The theme associated with quality of life factors was listed as an *increase in autonomy* (increase in locus of control, increase in autonomy, increase in responsibility, increase in motivation to self-manage, and increase in independence), with 18 of 118 (15%) occurrences [29,34,35,46,48,52-54,56,62,67,75,76]. One theme was associated with an *increase in cognitive ability* (increase in skill ability, increase in sensory organization, increase in memory, increase in cognitive activity, and increase in reasoning), with 11 of 118 (9%) occurrences [19,20,23,32,41,42,60]. Another theme was associated with a *decrease in symptoms surrounding diabetes* (decrease in HbA_1c_, decrease in cholesterol, increase in glucose management, and increase in diabetes health), with 9 of 118 (8%) occurrences [28,36,37,51,52,66]. Another theme was associated with an *increase in mindfulness of the condition* (increase in medical events detected, increase in education exposure, and more awareness of danger areas for falls like outside or stairwells), with 8 of 118 (7%) occurrences [21,27,38,43,47,51,63,67]. The next theme summarized observations of an *increase in the sense of safety, security, or comfort*, with 7 of 118 (6%) occurrences [21,23,44,47,53]. The last set of themes comprised 25% of the observations, and they were an *increase in health-related quality of life* (increase in life satisfaction and increase in the feeling of being understood); *increase in activity or coordination* (increase in mobility, increase in activity, increase in exercise, decrease in weight, decrease in BMI, increase in balance evaluation, and increase in the feeling of stability); *decrease in medical conditions surrounding pain* (decrease in alcohol abuse, decrease in cannabis misuse, decrease in cannabis dependence, and decrease in pain); *increase in documentation to give to the provider* (documentation and communication with the provider); *increase in satisfaction* (satisfaction with the health care system); and *increase in hospital metrics* (decrease in readmissions and decrease in hospital visits). The last observation was the only negative outcome. One participant noted that the SMS text messages she received as part of an exercise RCT *increased her level of guilt* because she was not exercising.

**Table 5:** Affinity matrix for medical outcomes and quality of life factors observed by older adults using telehealth.

Theme or observation	References	Number of occurrences (N=118)	Probability of occurrence
Decrease in psychological distress	[32,42,46,51,60,68,70,71,76]	21	19%
Increase in autonomy	[29,34,35,46,48,52-54,56,62,67,75,76]	18	16%
Not reported	[14-16,30,33,37-40,49,55,57-59,63-65,74]	18	16%
Increase in cognitive ability	[27,28,31,41,50,51,54,69]	11	10%
Decrease in medical conditions surrounding diabetes	[36,45,46,60,61,75]	9	8%
Increase in mindfulness of the condition	[29,35,47,52,56,60,72,77]	8	7%
Increase in safety or security	[29,31,53,56,62]	7	6%
Increase in health-related quality of life	[29,32,44,53,67,69]	6	5%
Increase in activity or coordination	[41,52,54,60]	6	5%
Decrease in medical conditions surrounding pain	[42,43,66]	5	4%
Increase in documentation to give the provider	[19,47,73]	3	3%
Increase in satisfaction	[4,5,53]	3	3%
Increase in hospital metrics	[26]	2	2%
Increase in guilt	[60]	1	1%

### Barriers

Fourteen themes and one observation that did not fit into a theme for barriers were observed. Table 6 lists the barriers with their associated references, number of occurrences, and probability of occurrence in this review. The barrier that was reported most often was *technical literacy* (I do not understand technology, I cannot navigate menus, I do not know how, etc) [14-16,19,27,28,34,35,37-40,46,47,49,56,58,59,63-65,75]. The theme noted the second most often was *lack of desire* (laziness, I do not want to, I am too busy, etc) [19,28,34,37, 38,49,57,58,60,63,64,67,74,76]. Another theme was *cost* (too expensive, we live off a fixed income, etc) [33,38, 40,48-50,55,58,63,64,67]. The theme *lack of technical support* included the following: my friends or family are not able to help me, I do not understand the interface, etc [14,28,34,58,63-65,73,74]. The theme *visual acuity* included the following: fonts or icons are too small, color contrast, etc [4,27,29,37,59,61,73]. The next observation was a surprise to our reviewing team; the theme was *social implications of using a telemonitoring* *device* (I do not want to bother a first responder, I do not want a stranger coming to my house, I do not want anyone coming to my house late at night, I had a bad experience the last time I used the telemonitoring device, I do not want my neighbor to overhear me using this thing, I do not have my own email, I do not understand social media, etc) [14,28,29,49,56,58,64,66]. The next theme was *ownership of technology* (no phone, no computer, no internet access, etc) [5,19,28,34,38,58,76]. The last set of themes and observations comprised less than 25% of the observations, and they were *privacy and security concerns*, *medical literacy* (I do not understand terminology, I do not understand test results, etc), *trust of the internet*, *mental acuity* (computers confuse me, the interface is too complex, I cannot focus for very long, how did I get to this page? etc), *hand-eye coordination* (particularly with those who have Parkinson disease, but not exclusively), *auditory acuity*, and *computer anxiety*.

**Table 6:** Affinity matrix for barriers to the use of telehealth by older adults.

Themes of barriers	References	Number of occurrences (N=144)	Probability of occurrence
Technical literacy	[14-16,19,27,28,34,35,37-40,46,47,49,56,58,59,63-65,75]	25	17%
Not reported	[26,31,32,36,41-45,51-54,62,68-72]	19	13%
Lack of desire	[19,28,34,37,38,49,57,58,60,63,64,67,74,76]	15	10%
Cost	[33,38,40,48-50,55,58,63,64,67]	11	8%
Lack of technical support	[14,28,34,58,63-65,73,74]	10	7%
Visual acuity	[4,27,29,37,59,61,73]	10	7%
Social implications	[14,28,29,49,56,58,64,66]	9	6%
Ownership of technology	[5,19,28,34,38,58,76]	8	6%
Privacy and security	[5,19,28,34,38,58,76]	8	6%
Medical literacy	[5,14,15,35,38,49,56,73]	8	6%
Trust of the internet	[5,14-16,30,58]	6	4%
Mental acuity	[27,29,38,59,63,73]	6	4%
Hand-eye coordination	[37,58,59,73]	4	3%
Auditory acuity	[4,50,61]	4	3%
Computer anxiety	[65]	1	1%

### Interactions Between Observations

There were several interactions worth discussing. We analyzed the interactions between interventions and barriers. Ten instances of eHealth interventions were mentioned with *technical literacy* [14-16,30,33,37,40,58,59,65,74]. Eight instances of eHealth interventions were mentioned with *lack of technical support* [14,28,34,58,59,65,73,74]. There were eight instances of mHealth interventions associated with *technical literacy* [19,27,28,35,38,39,49,64], but these were hardly mentioned at all with *lack of technical support* [28,74]. The interventions of mHealth were also associated with the barrier of *lack of desire*. This occurred six times in the literature [28,38,39,60,64,74]. Contrary to literature on the digital divide, eHealth and mHealth were only marginally associated with *ownership of technology*, which occurred four [5,28,34,58] and three times [19,28,38], respectively. Commensurate with literature on generational trends, both eHealth and mHealth were associated with *privacy and security concerns*, which occurred four [14,15,34,35] and two times [28,64], respectively. Both eHealth and mHealth were associated with the barrier *medical literacy*, which occurred four [5,14,15,73] and three times [35,38,39], respectively. Surprisingly, eHealth was associated with *hand-eye coordination*, but mHealth was not [37,58,60,73]. Finally, eHealth was associated with *lack of trust of the internet*, which occurred six times in the literature [5,14-16,30,58].

We also analyzed the interactions between interventions and medical outcomes. eHealth interventions were associated with an increase in cognitive ability. This interaction occurred seven times in the literature [28,31,41,50,51,54,69].

### Results Summary

This review identified 13 themes and one lone observation of medical outcomes incident with the adoption of five types of telehealth approaches. This review also identified 14 themes and one observation of barriers to the adoption of telehealth.

## Discussion

### Common Barriers to Telehealth

In this review, we were able to identify the common barriers associated with older adults utilizing telehealth. The most frequent barriers were lack of desire, cost, lack of technical support, visual acuity, social implications of use, ownership of technology, privacy and security, medical literacy, trust of the internet, mental acuity, hand-eye coordination, auditory acuity, and computer anxiety. Each of these barrier areas could present hurdles for elderly people dealing with telehealth and reasons to not use it. Lack of technical literacy is a large area of concern, as many elderly people have issues using computers to check email or smartphones to make telephone calls [13]. Because this is new to this population, they are also being held back from acceptance by a simple lack of wanting to do it [28,34,37-39,57,58,60,63,64,67,74,76]. It seems to be an easy thing to add to one’s daily tasks, but when one has lived largely without the use of these technologies, it can become an arduous task to “sell” the benefits of the sudden use of new technology and learning how to use new technology. They have the attitude “as it was not needed before, why bother to learn it now?” This can prove to be an uphill battle for providers who are attempting to utilize new technologies in different ways.

The cost of technology is also quite prohibitive, as computers, smartphones, and other devices cost hundreds to thousands of dollars. Those living on fixed incomes are cash strapped and may not be able to afford to purchase or use such new technologies. Not owning such technologies presents its own concerns for the provision of care. Besides cost, there are concerns in this population regarding the ability to actually utilize the modality of telehealth efficiently. Issues with visual acuity [4,27,29,37,59,61,73], mental acuity [27,29,38,59,63,73], hand-eye coordination [37,58,59,73], and auditory acuity [4,50,61] are all relevant concerns for elderly people. Many people, as they become older, experience decreases in the efficiencies of the operations of many body systems, including their senses. Many develop disease processes that can affect their mental status, vision, and hearing, and any or all of these could easily lead to problems with being able to use technology, let alone having a clear understanding of what they need to be doing with the device or even how to interact with it.

The elderly population also has relevant concerns with trust and technology, as they are one of the prime targets for abuse from their use of technology according to popular media [13,78]. This is where lack of technical support for the use of technology can become a very relevant area of concern. There is no affordable and adequate source of “technical support” to simply learn how to use devices [14,28,59,73]. This lack of knowledge and available education can be a very problematic barrier for the use of the modality of telehealth. Furthermore, problems surrounding trust of the internet [5,14-16,30,58], concerns of privacy and security [5,19,28,34,38,58,76], and even computer anxiety [65] can figure into the use of technology. As there are concerns with privacy and security, telehealth could easily cause patients to succumb to some level of anxiety. Not understanding the modality of telehealth or how to use it can add to the level of this anxiety at an exponential rate.

Another consideration with the use of telehealth is that it requires a certain level of user knowledge. The utilization of medical applications requires the user to have some knowledge of medical terms, procedures, etc [5,14,15,35,38,49,56,73]. This is often not the case, as this population was raised without the internet or medical knowledge. Medical knowledge came from physicians during their younger years, and only recently, the approach has changed to the utilization of internet web searches to garner knowledge about symptoms and diagnoses. This is an entirely new world for the elderly population and a relevant barrier to the use of these applications overall. Overcoming this knowledge gap could prove to be an insurmountable task or one that requires any telehealth use to be kept to an absolute minimum for knowledge or know-how on the part of the user.

### Common Outcomes Associated With Telehealth Interventions

The research supports strong medical outcomes incident to the use of telehealth as follows: *decreased psychological distress* [32,42,46,51,60,68,70,71,76], *increased autonomy* [29,34,35,46,48,52-54,56,62,67,75,76], *increased cognitive ability* [27,28,31,41,50,51,54,69], and many others. This review supports an *increased quality of life* for those who adopt telehealth [29,32,44,53,67,69]. The use of telehealth can lead to less psychological distress, as users know that they have a way of communicating their medical concerns to their providers in a much easier and faster way. This could eventually enable better health due to better management, thus allowing for fewer associated medical conditions for those patients who use telehealth for assistance in the management of their care.

The observation of greater documentation for providers demonstrated that the use of telehealth is not all about the patient. It is just as much about practitioners providing care. The use of telehealth allows for much faster accessibility to documentation to provide care or even real-time information about the patient to allow for immediate diagnosis or intervention, based on information being gathered by the used technology. This can make the provision of care easier and much more efficient for the field, which is already seeing more patients than it can comfortably manage.

### Interactions Among Outcomes, Barriers, and Types of Interventions

eHealth interventions were the most frequently observed interventions in the literature, and these interventions were most frequently associated with the barriers of technical literacy and lack of technical support. This observation is interesting because general technical support, whether from friends, neighbors, family, or caregivers, or professionally acquired technical support is a control for the barrier of technical literacy. The interaction between eHealth and *technical literacy* is interesting as well. This could signal that older adults are more adept at mobile technology than computer technology for application of telehealth. This supposition is supported by the literature because many older adults are turning to mobile technology to communicate with children and grandchildren [13]. The interaction between mHealth and *lack of desire* is noteworthy. This seems to indicate that older adults are willing to interact with mobile technology to communicate with children and grandchildren, but they are not as willing to use it for telehealth interventions.

### Study Quality and Literature Bias

The assessment of the quality of the articles studied is worthy of discussion. The majority (27/57, 49%) of the articles analyzed were level III (nonexperimental, qualitative, or meta-synthesis studies). The reviewers would have preferred to analyze only the highest level (level I) (experimental study or RCT), but only 10 (17%) such studies were available. Fortunately, 98% (56/57) of the articles were rated as quality level A (high quality) or B (good quality). The importance of this rating cannot be understated. If the findings from this review were from low-quality articles, the results would not be as strong. By analyzing high-quality articles with strong levels of evidence, readers can be more assured of the results. Research articles with strong study designs and sufficiently large samples are generally accepted in the scientific field for their veracity.

### Limitations

The authors identified the low number of articles analyzed as a limitation of this systematic review. If the authors conduct another systematic review on the same topic, they would like to have a larger analysis pool. This could be achieved by broadening the years of study in the selection or by reducing the threshold of quality. However, the additional years of study would only repeat the results from previously published reviews of a similar topic, and lowering the threshold of quality would introduce articles with dubious results.

Although not intentional, the authors realized that selection bias may be present in this article. To combat selection bias, the authors worked to minimize its effects by ensuring each article was reviewed by at least two authors. The authors held consensus meetings after each screening to provide feedback and reach total agreement on the inclusion and exclusion of articles for the analysis.

Another source of bias that could have affected this article is publication bias. To control for publication bias, the authors searched the Boolean search string in Google Scholar. This action was intended to identify articles from lesser-known journals that may not have appeared in MEDLINE or CINAHL.

Another limitation is our inclusion of people aged 50 years or above in the study of older adults. Most studies categorize older adults as those aged 65 years or above. The elderly population currently spans baby boomers and the silent generation. The youngest members of the former group are still working and are most likely using technology fluently. It is possible that our generalizations do not apply to all members of the elderly demographic.

### Future Research

Health care systems can utilize knowledge of these barriers to develop solutions for broadening the use of telehealth among older adults. A multidisciplinary approach and culture of collaboration between administrative leadership and providers may be the most effective and immediate manner of implementing solutions to breach these barriers and strengthen the reach of health care services. However, some barriers may be out of the scope of impact, and policy makers should consider supporting the efforts. Future research should be conducted on methods for personalizing telehealth in older adults before implementation. 

### Conclusion

Providing sufficient health care access to the rapidly growing aging population has been an imminent issue, and telehealth is a useful tool that can provide a solution. While health care systems increase their telehealth efforts to improve access to health care services among vulnerable populations, such as older adults, some health care organizations do not consider the technological, educational, financial, and behavioral barriers before implementing telehealth solutions. It is imperative that health care systems use a multidisciplinary approach and collaborate with health care providers, community partners, and policy makers to address these barriers of utilizing telehealth among older adults and to successfully implement telehealth solutions. This systematic review provides some understanding of older adults’ perspectives and experiences with the barriers of implementing telehealth services.

## Appendix

Multimedia Appendix 1 Detailed observations on interventions and corresponding themes.

Multimedia Appendix 2 Detailed observations on medical outcomes and corresponding themes.

Multimedia Appendix 3 Detailed observations on barriers and corresponding themes.

Multimedia Appendix 4 Bias, country of origin, statistics, and quality assessments.

## Abbreviations

JHNEBP: John Hopkins Nursing Evidence-Based Practice
MeSH: Medical Subject Headings
mHealth: mobile health
RCT: randomized controlled trial
WHO: World Health Organization
