# Supplementary material for: Utilization Barriers and Medical Outcomes Commensurate With the Use of Telehealth Among Older Adults: Systematic Review
Source: JMIR Med Inform. 2020 Aug 12;8(8):e20359. doi: 10.2196/20359 (PMC7450384; doi:10.2196/20359)
Supplement: Multimedia Appendix 1 [file medinform_v8i8e20359_app1.docx]

**Appendix A: Table of detailed observations on interventions and corresponding themes**

| Authors | Intervention | Intervention Theme |  |
| --- | --- | --- | --- |
|  |  |  |  |
| Hamilton et al 2020 | Tele-monitoring Remote patient monitoring (RPM): Blood-pressure cuffs, pulse oximeters, body weight scales Telehealth Intervention Programs for Seniors (TIPS), remote patient monitoring (RPM), extensive social wraparound services, care coordination, and intergenerational socialization aimed at improving health care options to assist low-income, high health-risk older adults who live in subsidized congregate housing or attend local community centers for older adults.  A survey instrument was collected each week. | Tele-monitoring |  |
| Theis et al 2019 | eHealth | eHealth |  |
| Wildenbos et al 2019 | mHealth Investigated these interaction issues in two different case studies: an app for older adults facilitating their hospital appointment attendance (App 1) and a self-monitoring app for chronically ill older patients | mHealth |  |
| Jakobsson et al 2019 | Telehealth Smartphone, Computer, Landline | mHealth |  |
|  |  | eHealth |  |
|  |  | Telecare (phone) |  |
| Karlsen et al 2019 | Tele-monitoring Personal alarm (16), light sensors (3), stove alarm (4), GPS tracking (3), medication reminders (8), bed sensors (1), door sensor 2), video surveillance (2) | Tele-monitoring |  |
|  |  | Video call |  |
| Coley et al 2019 | eHealth Participants were randomized to either an interactive Internet platform, designed to encourage goal setting and lifestyle changes with the remote support of a lifestyle coach, or a control platform with basic health information but no interactive features or coach support. Owing to the nature of the intervention, complete double blinding was not possible, but masking was attempted by informing participants only that they would be randomized to one of 2 Internet platforms (without further details on their content). | eHealth |  |
| Giesbrecht & Miller 2019 | eHealth The treatment group incorporated two in-person training sessions with a trainer and 4 weeks of monitored home training using a computer tablet (mHealth) wheelchair skills program. The control group did not receive MWC skills training, as is typical practice with this population | eHealth |  |
| Brodbeck 2019 | eHealth Internet-based self-help intervention for prolonged grief symptoms after spousal bereavement or separation/divorce | eHealth |  |
| Mosley et al 2019 | eHealth Etymotic Home Hearing Test (HHT) compared with traditional, manual audiometry (MA) | eHealth |  |
| Jensen et al 2019 | eHealth "My Hip Fracture Journey" on iPad (provided) education through pictographs, video clips, illustrated exercises and written information. This was used to augment home visits and subsequent interviews. | eHealth |  |
| Rasche et al 2018 | eHealth The national survey queried the use of health apps and their perceived usefulness. | eHealth |  |
| Portz et al 2018 | mHealth The HF app was developed to allow patients to track their symptoms of HF. Thirty (N = 30) older adults completed an acceptability survey after using the mobile app. The survey used Likert items and open-ended feedback questions. | mHealth |  |
| Castro et al 2018 | eHealth Participants were matched into geographically based small groups with an assigned health coach and they began the program at the same time. Group members were connected to each other through a private online social forum where they could post comments and questions, engage in health coach–moderated discussions, and provide social support to one another. Using Internet-enabled devices (laptop, tablet, or smartphone), program participants were able to asynchronously complete weekly interactive curriculum lessons. reflections and goal-setting activities in relation to the weekly topic | eHealth |  |
| Joe et al 2018 | eHealth A focus-group method was used to brainstorm designs for telehealth for older adults. | eHealth |  |
| Dham et al 2018 | Telehealth Telepsychiatry assessments | Video call |  |
| Paige et al 2018 | eHealth eHealth awareness and eHealth literacy scale | eHealth |  |
| Cajita MI, et al 2018 | mHealth | mHealth |  |
| Harte et al 2018 | mHealth training on smartphone -based fall detection and prevention system | mHealth |  |
| Gordon & Hornbrook 2018 | eHealth online forms, online tracking systems, patient portal |  |  |
| Bao et al 2018 | eHealth Online training | eHealth |  |
| Egede et al 2018 | Telehealth Telepsychotherapy | Video call |  |
| Platts-Mills et al 2018 | Telecare Telephone call, protocol-guided follow up | Telecare (phone) |  |
| Lopez-Villegas et al 2018 | Tele-monitoring pacemakers | Tele-monitoring |  |
| Dugas et al 2018 | mHealth DiaSocial for glucose control, exercise, nutrition, and medication adherence | mHealth |  |
| Nalder et al 2018 | eHealth Three Internet-based platforms 1. Chronic disease management  2. Real-world strategy training 3. Learning the ropes | eHealth |  |
| Buck et al 2017 | eHealth (PSHA) a web-based, tablet delivered intervention, developed internally, which encourages the participant to record their daily medication intake, weight and time spent with a brief exercise program using an aerobic stepper. Tablets record daily information and watch one short heart health educational video. | eHealth |  |
| Ware et al 2017 | eHealth | eHealth |  |
| Chang et al 2017 | Telehealth Diabetes management | mHealth |  |
| Cajita 2017 | mHealth Simple linear regression was used to test the relationship between the main study variables (eHealth literacy, social influence, perceived financial cost, perceived ease of use, and perceived usefulness) and intention to use mHealth. | mHealth |  |
| LaMonica 2017 | eHealth memory aids, mental acuity exercises, | eHealth |  |
| Bahar-Fuchs 2017 | eHealth Tailored and adaptive computer cognitive training in older adults at risk for dementia | eHealth |  |
| Nahm et al 2017 | eHealth Bone Power program | eHealth |  |
| Knaevelsrud et al 2017 | eHealth Internet-based therapist-guided intervention | eHealth |  |
| Reijnders et al 2017 | eHealth cognitive functioning | eHealth |  |
| Mageroski et al. 2016 | Tele-monitoring Remote sensors in homes of older adults | Tele-monitoring |  |
| Hamblin et al 2016 | Tele-monitoring | Tele-monitoring |  |
| Wang et al 2016 | Tele-monitoring Wearables, mobile devices, trackers, in-home telemonitoring | Tele-monitoring |  |
| Gordon & Hornbrook 2016 | eHealth | eHealth |  |
| Williams et al 2016 | eHealth | eHealth |  |
| Evans J, et al 2016 | mHealth remote monitoring, wrist wearable and wireless tablet | mHealth |  |
| Muller et al 2016 | mHealth short message service (SMS), Physical Activity for Health Study (PAtHS) | mHealth |  |
| Quinn et al 2016 | mHealth mobile diabetes intervention study (MDIS) | mHealth |  |
| Royackers et al 2016 | eHealth point of care technology through eShift (home-based palliative care) | eHealth |  |
| Duh et al 2016 | Telecare CareMe | Telecare (phone) |  |
| Depatie et al 2015 | mHealth mobile health technology for older adults in rural communities | mHealth |  |
| Moore et al 2015 | eHealth Internet-based hearing health care for older adults | eHealth |  |
| Currie et al 2015 | eHealth | eHealth |  |
| Grant et al 2015 | Tele-monitoring LivingWell@Home, sensors (motion, bed, humidity), emergency response systems, biometric monitors (heart rate, blood pressure, weight, pulse oximetry, blood glucose) | Tele-monitoring |  |
| Brenes GA et al 2015 | Telecare Telephone-delivered cognitive behavior therapy and telephone-delivered nondirective supportive therapy | Telecare (phone) |  |
| Corbett et al 2015 | eHealth online cognitive training package | eHealth |  |
| Mavandadi S et al 2015 | Telecare SUSTAIN care management system (assessment, monitoring, care management, and brief therapies) | Telecare (phone) |  |
| Egede et al 2015 | Telehealth Tele-psychotherapy | Video call |  |
| Chang W et al 2015 | Tele-monitoring Remote cardiology management | Tele-monitoring |  |
| Boulos et al 2015 | eHealth LiveWell Parkinson's intervention, learning modules | eHealth |  |
| Dino & deGuzman 2015 | Telehealth, mHealth, eHealth | mHealth |  |
|  |  | eHealth |  |
|  |  | Tele-monitoring |  |
| Czaja et al 2015 | Tele-monitoring Telehealth system that monitors blood pressure and bodyweight | Tele-monitoring |  |
| Choi NG et al 2015 | Telecare Video tele-problem-solving therapy (PST) to in-person PST and telephone care calls | Video call |  |
