# Supplementary material for: Utilization Barriers and Medical Outcomes Commensurate With the Use of Telehealth Among Older Adults: Systematic Review
Source: JMIR Med Inform. 2020 Aug 12;8(8):e20359. doi: 10.2196/20359 (PMC7450384; doi:10.2196/20359)
Supplement: Multimedia Appendix 2 [file medinform_v8i8e20359_app2.docx]

**Appendix B: Table of detailed observations on medical outcomes and corresponding themes**

| Authors | Medical Outcomes Reported | Medical Outcome Theme |  |
| --- | --- | --- | --- |
|  |  |  |  |
| Hamilton T, et al 2020 | Decrease in hospital visits,  Decrease in readmissions, | Increase in Hospital metrics |  |
|  |  | Increase in Hospital metrics |  |
| Theis S, et al 2019 | Increase in satisfaction,  64% older adults are satisfied with the health information they receive, 34% are neutral, 2% dissatisfied | Increase in satisfaction |  |
|  |  |  |  |
|  |  |  |  |
|  |  |  |  |
| Wildenbos GA, et al 2019 | Decrease in Cognitive impairment was reported but not compared with a control | Increase in cognitive ability |  |
|  |  |  |  |
|  |  |  |  |
|  |  |  |  |
|  |  |  |  |
| Jakobsson E, et al 2019 | Decrease in Cognitive impairment was reported but not compared with a control | Increase in cognitive ability |  |
|  |  |  |  |
|  |  |  |  |
|  |  |  |  |
|  |  |  |  |
|  |  |  |  |
|  |  |  |  |
| Karlsen C, et al 2019 | Increase in safety,  Increase in satisfaction,  Increase in security,  Increase in independence,  Increase in responsibility,  Increase in mindfulness of frailty | Increase in safety or security |  |
|  |  | Increase in health-related quality of life |  |
|  |  | Increase in safety or security |  |
|  |  | Increase in safety or security |  |
|  |  | Increase in autonomy |  |
|  |  | Increase in mindfulness of condition |  |
| Coley N, et al 2019 | Not reported | Not reported |  |
| Giesbrecht & Miller 2019 | Increase in skill capacity,  Increase in safety | Increase in cognitive ability |  |
|  |  | Increase in safety or security |  |
| Brodbeck J, et al 2019 | Decrease in grief,  Decrease in depression,  Decrease in psychological distress,  Decrease in embitterment,  Decrease in loneliness,  Increase in life satisfaction | Decrease in psychological distress |  |
|  |  | Decrease in psychological distress |  |
|  |  | Decrease in psychological distress |  |
|  |  | Decrease in psychological distress |  |
|  |  | Decrease in psychological distress |  |
|  |  | Increase in health-related quality of life |  |
| Mosley C, et al 2019 | Not reported | Not reported |  |
| Jensen C, et al. 2019 | Increase in autonomy,  Increase in self-care | Increase in autonomy |  |
|  |  | Increase in autonomy |  |
|  |  |  |  |
|  |  |  |  |
|  |  |  |  |
| Rasche P, et al 2018 | Not reported | Not reported |  |
|  |  |  |  |
|  |  |  |  |
|  |  |  |  |
|  |  |  |  |
|  |  |  |  |
| Portz JD, et al 2018 | Increase in awareness of condition,  Increase in self-care | Increase in mindfulness of condition |  |
|  |  | Increase in autonomy |  |
| Castro Sweet CM, et al 2018 | Decrease in weight participants lost an average of 13 to 14 pounds (8%) Decrease in HbA1c 0.14% absolute decrease at 6 months and 12 months (*p* = .0001) Decrease in cholesterol mean reduction of -12.92mg/dL (*p* = .0001) | Decrease in medical conditions surrounding diabetes |  |
|  |  | Decrease in medical conditions surrounding diabetes |  |
|  |  | Decrease in medical conditions surrounding diabetes |  |
| Joe J, et al 2018 | Not reported | Not reported |  |
|  |  |  |  |
|  |  |  |  |
|  |  |  |  |
|  |  |  |  |
|  |  |  |  |
| Dham P, et al 2018 | High satisfaction | Increase in satisfaction |  |
|  |  |  |  |
| Paige SR, et al 2018 | Not reported | Not reported |  |
|  |  |  |  |
| Cajita MI, 2018 | Not reported | Not reported |  |
|  |  |  |  |
|  |  |  |  |
|  |  |  |  |
|  |  |  |  |
|  |  |  |  |
| Harte R, 2018 | Not reported | Not reported |  |
| Gordon NP & Hornbrook MC 2018 | Not reported | Not reported |  |
|  |  |  |  |
| Bao T, et al 2018 | Increase in sensory organization test, Increase in Mini balance evaluation system test, Increase in five times sit to stand test, no statistical significance in other clinical outcomes | Increase in cognitive ability |  |
|  |  | Increase in activity or coordination |  |
|  |  | Increase in activity or coordination |  |
| Egede LE, et al 2018 | Decrease in baseline depression severity, Decrease in generalized anxiety disorder, Decrease in alcohol misuse, Decrease in cannabis misuse, Decrease in cannabis dependence | Decrease in psychological distress |  |
|  |  | Decrease in psychological distress |  |
|  |  | Decrease in medical conditions surrounding pain |  |
|  |  | Decrease in medical conditions surrounding pain |  |
|  |  | Decrease in medical conditions surrounding pain |  |
| Platts-Mills TF, 2018 | Decrease in pain | Decrease in medical conditions surrounding pain |  |
| Lopez-Villegas A, et al 2018 | Increase in EQ-5D VAS (health related quality of life) | Increase in health-related quality of life |  |
| Dugas M, et al 2018 | Increase in glucose management, Decrease in HbA1c | Decrease in medical conditions surrounding diabetes |  |
|  |  | Decrease in medical conditions surrounding diabetes |  |
| Nalder E, et al 2018 | Decrease in HbA1c, Increase in independence, Increase in emotional support, Increase in motivation to self-manage | Decrease in medical conditions surrounding diabetes |  |
|  |  | Increase in autonomy |  |
|  |  | Decrease in psychological distress |  |
|  |  | Increase in autonomy |  |
| Buck H, et al 2017 | Increase in documentation for nutrition, eating, Increase in instructional video exposure | Increase in documentation to give provider |  |
|  |  | Increase in mindfulness of condition |  |
| Ware P, et al 2018 | Not reported | Not reported |  |
|  |  |  |  |
|  |  |  |  |
|  |  |  |  |
|  |  |  |  |
|  |  |  |  |
| Chang CP, et al 2017 | Increase in self-management, Increase in independence | Increase in autonomy |  |
|  |  | Increase in autonomy |  |
| Cajita MI, et al 2017 | Not reported | Not reported |  |
|  |  |  |  |
|  |  |  |  |
|  |  |  |  |
|  |  |  |  |
| LaMonica HM, 2017 | Increase in memory | Increase in cognitive ability |  |
|  |  |  |  |
|  |  |  |  |
| Bahar-Fuchs A, et al 2016 | Increase in memory, Increase in global cognition, Increase in learning, Increase in mood | Increase in cognitive ability |  |
|  |  | Increase in cognitive ability |  |
|  |  | Increase in cognitive ability |  |
|  |  | Decrease in psychological distress |  |
| Nahm ES, et al 2016 | Increase in osteoporosis knowledge, Increase in self-efficacy/outcome expectations, Increase in exercise behaviors | Increase in mindfulness of condition |  |
|  |  | Increase in autonomy |  |
|  |  | Increase in activity or coordination |  |
| Knaevelsrud C, et al 2016 | Increase in comfort (from not being able to see the therapist), Increase in satisfaction, Increase in motivation, Increase in feeling of being understood | Increase in safety or security |  |
|  |  | Increase in satisfaction |  |
|  |  | Increase in autonomy |  |
|  |  | Increase in health-related quality of life |  |
| Reijnders JS, et al 2016 | Increase in feelings of stability, Increase in memory functioning, Increase in locus of control | Increase in activity or coordination |  |
|  |  | Increase in cognitive ability |  |
|  |  | Increase in autonomy |  |
| Mageroski A, et al 2016 | Not reported | Not reported |  |
|  |  |  |  |
| Hamblin K, et al 2016 | Increase in autonomy,  Increase in awareness of danger areas like gardens or staircases, Increase in safety | Increase in autonomy |  |
|  |  | Increase in mindfulness of condition |  |
|  |  | Increase in safety or security |  |
|  |  |  |  |
| Wang J, et al 2016 | Not reported | Not reported |  |
| Gordon NP & Hornbrook MC 2016 | Not reported | Not reported |  |
|  |  |  |  |
|  |  |  |  |
|  |  |  |  |
|  |  |  |  |
|  |  |  |  |
|  |  |  |  |
|  |  |  |  |
| Williams K, et al 2016 | Not reported | Not reported |  |
|  |  |  |  |
|  |  |  |  |
|  |  |  |  |
|  |  |  |  |
| Evans J, et al [12] | Increase in documentation for weight, blood pressure | Increase in documentation to give provider |  |
|  |  |  |  |
|  |  |  |  |
|  |  |  |  |
| Muller AM, et al 2016 | Increase in exercise, Increase in mood, Increase in fitness, Increase in health, Increase in mindfulness of the importance of exercise, Increase in guilt | Increase in activity or coordination |  |
|  |  | Decrease in psychological distress |  |
|  |  | Increase in activity or coordination |  |
|  |  | Decrease in medical conditions surrounding diabetes |  |
|  |  | Increase in mindfulness of condition |  |
|  |  | Increase in guilt |  |
| Quinn CC, et al 2016 | Decrease in HbA1c | Decrease in medical conditions surrounding diabetes |  |
|  |  |  |  |
| Royackers A, et al 2016 | Increase in comfort, Increase in independence, Increase in autonomy | Increase in safety or security |  |
|  |  | Increase in autonomy |  |
|  |  | Increase in autonomy |  |
| Duh E, et al 2016 | Not reported | Not reported |  |
|  |  |  |  |
|  |  |  |  |
|  |  |  |  |
|  |  |  |  |
| Depatie, A & Bigbee, JL 2015 | Not reported | Not reported |  |
|  |  |  |  |
|  |  |  |  |
|  |  |  |  |
|  |  |  |  |
|  |  |  |  |
| Moore AN, et al 2015 | Not reported | Not reported |  |
|  |  |  |  |
|  |  |  |  |
| Currie M, et al 2015 | Decrease in pain | Decrease in medical conditions surrounding pain |  |
| Grant LA, et al 2015 | Increase in satisfaction, Increase in autonomy, Increase in independence | Increase in health-related quality of life |  |
|  |  | Increase in autonomy |  |
|  |  | Increase in autonomy |  |
| Brenes GA, et al 2015 | Decrease in worry, Decrease in GAD, Decrease in depression, Decrease in anxiety | Decrease in psychological distress |  |
|  |  | Decrease in psychological distress |  |
|  |  | Decrease in psychological distress |  |
|  |  | Decrease in psychological distress |  |
| Corbett A, et al 2015 | Increase in reasoning, Increase in verbal learning, Increase in IADL | Increase in cognitive ability |  |
|  |  | Increase in cognitive ability |  |
|  |  | Increase in health related quality of life |  |
| Mavandadi S, et al 2015 | Decrease in depressive symptoms, Decrease in anxiety symptoms, Increase in MH Functioning | Decrease in psychological distress |  |
|  |  | Decrease in psychological distress |  |
|  |  | Decrease in psychological distress |  |
| Egede LE, et al 2015 | Decrease in GDS, Decrease in BDI, Decrease in DSM-IV | Decrease in psychological distress |  |
|  |  | Decrease in psychological distress |  |
|  |  | Decrease in psychological distress |  |
| Chang W, et al 2015 | Increase in cardiac arrhythmias detected, Increase in paroxysmal atrial fibrillation detected | Increase in mindfulness of condition |  |
|  |  | Increase in mindfulness of condition |  |
| Boulos M, et al 2015 | Increase in communication of condition with provider | Increase in documentation to give provider |  |
|  |  |  |  |
|  |  |  |  |
|  |  |  |  |
|  |  |  |  |
| Dino M & deGuzman A 2015 | Not reported | Not reported |  |
|  |  |  |  |
|  |  |  |  |
| Czaja SJ, et al 2015 | Increase in self-management, Increase in health, Increase in independence | Increase in autonomy |  |
|  |  | Decrease in medical conditions surrounding diabetes |  |
|  |  | Increase in autonomy |  |
| Choi NG, et al 2015 | Decrease in depressive symptoms, Increase in understanding of depression, Increase in social interaction | Decrease in psychological distress |  |
|  |  | Increase in mindfulness of condition |  |
|  |  | Increase in autonomy |  |
