# Supplementary material for: Utilization Barriers and Medical Outcomes Commensurate With the Use of Telehealth Among Older Adults: Systematic Review
Source: JMIR Med Inform. 2020 Aug 12;8(8):e20359. doi: 10.2196/20359 (PMC7450384; doi:10.2196/20359)
Supplement: Multimedia Appendix 3 [file medinform_v8i8e20359_app3.docx]

**Appendix C: Table of detailed observations on barriers and corresponding themes**

| Authors | Barriers | Theme of Barriers |  |
| --- | --- | --- | --- |
|  |  |  |  |
| Hamilton T, et al 2020 | Not reported | Not reported |  |
|  |  |  |  |
| Theis S, et al 2019 | Medical literacy,  Trust of Internet information,  possession of computer, possession of smartwatch | Medical literacy |  |
|  |  | Trust of Internet |  |
|  |  | Ownership of technology |  |
|  |  | Ownership of technology |  |
| Wildenbos GA, et al 2019 | Reading small fonts, Information overload, Clicking small icons, Interacting with scroll bar, Lack of understanding of app capabilities | Visual acuity |  |
|  |  | Mental acuity |  |
|  |  | Visual acuity |  |
|  |  | Technical literacy |  |
|  |  | Technical literacy |  |
| Jakobsson E, et al 2019 | Fear of others overhearing, Security, Not aware of capabilities, No perceived value, Not in habit of using, No access to technology, Fear of loss of info, Need support to use | Social implications |  |
|  |  | Privacy and security |  |
|  |  | Technical literacy |  |
|  |  | Lack of desire |  |
|  |  | Ownership of technology |  |
|  |  | Privacy and security |  |
|  |  | Lack of tech support |  |
| Karlsen C, et al 2019 | Forget to wear tech, Difficulty with small fonts and icons, Desire a personal relationship with provider | Mental acuity |  |
|  |  | Visual acuity |  |
|  |  | Social implications |  |
|  |  |  |  |
|  |  |  |  |
|  |  |  |  |
| Coley N, et al 2019 | Unreliability of information on Internet | Trust of Internet |  |
| Giesbrecht & Miller 2019 | Not reported | Not reported |  |
|  |  |  |  |
| Brodbeck J, et al 2019 | Not reported | Not reported |  |
|  |  |  |  |
|  |  |  |  |
|  |  |  |  |
|  |  |  |  |
|  |  |  |  |
| Mosley C, et al 2019 | cost | Cost |  |
| Jensen C, et al. 2019 | Privacy, Ownership of technology, busy doing other things, Content with advice from peers, Difficulty with app | Privacy and security |  |
|  |  | Ownership of technology |  |
|  |  | Lack of desire |  |
|  |  | Lack of tech support |  |
|  |  | Technical literacy |  |
| Rasche P, et al 2018 | Lack of trust of apps, Lack of confidence using apps, Technical savvy, Computer literacy,  Health literacy, Privacy concerns | Trust of Internet |  |
|  |  | Technical literacy |  |
|  |  | Technical literacy |  |
|  |  | Technical literacy |  |
|  |  | Medical literacy |  |
|  |  | Privacy and security |  |
| Portz JD, et al 2018 | Difficulty navigating app, Health literacy | Technical literacy |  |
|  |  | Medical literacy |  |
| Castro Sweet CM, et al 2018 | Not reported | Not reported |  |
|  |  |  |  |
|  |  |  |  |
| Joe J, et al 2018 | Font size, color contrast, depth perception, hand-eye coordination, interface too complicated, perceived usefulness, | Visual acuity |  |
|  |  | Visual acuity |  |
|  |  | Visual acuity |  |
|  |  | Hand-eye coordination |  |
|  |  | Technical literacy |  |
|  |  | Lack of desire |  |
| Dham P, et al 2018 | Audio clarity, Visual clarity | Visual acuity |  |
|  |  | Auditory acuity |  |
| Paige SR, et al 2018 | Lack of technical ability, Trust of Internet resources | Technical literacy |  |
|  |  | Trust of Internet |  |
| Cajita MI, 2018 | Lack of understanding of mHealth, decreases sensory perception, lack of need of technology, poorly designed interface, cost of technology, limited/fixed income | Medical literacy |  |
|  |  | Mental acuity |  |
|  |  | Lack of desire |  |
|  |  | Technical literacy |  |
|  |  | Ownership of technology |  |
|  |  | Cost |  |
| Harte R, 2018 | Difficult interface | Technical literacy |  |
| Gordon NP & Hornbrook MC 2018 | cost, lack of self confidence | Cost |  |
|  |  | Technical literacy |  |
| Bao T, et al 2018 | Not reported | Not reported |  |
|  |  |  |  |
|  |  |  |  |
| Egede LE, et al 2018 | Not reported | Not reported |  |
|  |  |  |  |
|  |  |  |  |
|  |  |  |  |
|  |  |  |  |
| Platts-Mills TF, 2018 | Not reported | Not reported |  |
| Lopez-Villegas A, et al 2018 | Not reported | Not reported |  |
| Dugas M, et al 2018 | Not reported | Not reported |  |
|  |  |  |  |
| Nalder E, et al 2018 | technological problems | Technical literacy |  |
|  |  |  |  |
|  |  |  |  |
|  |  |  |  |
| Buck H, et al 2017 | technological problems | Technical literacy |  |
|  |  |  |  |
| Ware P, et al 2017 | difficulty identifying credible and meaningful sources on the Internet, confusion on ownership of medical information, Do not know how to access medical information, confusion about responsibility for medical information, Availability of peer support, privacy concerns | Trust of Internet |  |
|  |  | Medical literacy |  |
|  |  | Technical literacy |  |
|  |  | Social implications |  |
|  |  | Lack of tech support |  |
|  |  | Privacy and security |  |
| Chang CP, et al 2017 | Cost, | Cost |  |
|  |  |  |  |
| Cajita MI, et al 2017 | eHealth literacy, perceived usefulness, cost, difficult to use, social influence | Medical literacy |  |
|  |  | Lack of desire |  |
|  |  | Cost |  |
|  |  | Technical literacy |  |
|  |  | Social implications |  |
| LaMonica HM, 2017 | access to Internet, cost, lack skills | Auditory acuity |  |
|  |  | Cost |  |
|  |  | Auditory acuity |  |
| Bahar-Fuchs A, et al 2017 | Not reported | Not reported |  |
|  |  |  |  |
|  |  |  |  |
|  |  |  |  |
| Nahm ES, et al 2017 | Not reported | Not reported |  |
|  |  |  |  |
|  |  |  |  |
| Knaevelsrud C, et al 2017 | Not reported | Not reported |  |
|  |  |  |  |
|  |  |  |  |
|  |  |  |  |
| Reijnders JS, et al 2017 | Not reported | Not reported |  |
|  |  |  |  |
|  |  |  |  |
| Mageroski A, et al 2016 | cost, privacy & security | Cost |  |
|  |  | Privacy and security |  |
| Hamblin K, et al 2016 | mechanical problems, confusion about what would happen if the alarm is triggered for a fall,  not wanting to summon a stranger at night, negative experience the last time the alarm was triggered | Technical literacy |  |
|  |  | Medical literacy |  |
|  |  | Social implications |  |
|  |  | Social implications |  |
| Wang J, et al 2016 | Not interested in technology, | Lack of desire |  |
| Gordon NP & Hornbrook MC 2016 | Owning technology, No access, costs, unfamiliarity with the Internet, physical limitations to using technology, does not have an email account, do not know how to interact, unwilling to use technology | Ownership of technology |  |
|  |  | Lack of tech support |  |
|  |  | Cost |  |
|  |  | Technical literacy |  |
|  |  | Hand-eye coordination |  |
|  |  | Trust of Internet |  |
|  |  | Social implications |  |
|  |  | Lack of desire |  |
| Williams K, et al 2016 | unfamiliar with technology, confusing interface, make mistakes, difficulty seeing icons, hand-to-eye coordination | Technical literacy |  |
|  |  | Lack of tech support |  |
|  |  | Mental acuity |  |
|  |  | Visual acuity |  |
|  |  | Hand-eye coordination |  |
| Evans J, et al 2016 | no patience, complicated interface, do not want to wear the watch, no access to Internet, | Lack of desire |  |
|  |  | Technical literacy |  |
|  |  | Lack of desire |  |
|  |  | Ownership of technology |  |
| Muller AM, et al 2016 | laziness | Lack of desire |  |
|  |  |  |  |
|  |  |  |  |
|  |  |  |  |
|  |  |  |  |
|  |  |  |  |
| Quinn CC, et al 2016 | severe impairment of hearing or vision | Visual acuity |  |
|  |  | Auditory acuity |  |
| Royackers A, et al 2016 | Not reported | Not reported |  |
|  |  |  |  |
|  |  |  |  |
| Duh E, et al 2016 | lack of focus, perceived usefulness, difficulty understanding technology, complicated interfaces, cost | Mental acuity |  |
|  |  | Lack of desire |  |
|  |  | Lack of tech support |  |
|  |  | Technical literacy |  |
|  |  | Cost |  |
| Depatie, A & Bigbee, JL 2015 | cost, not interested, not comfortable communicating with provider that way, ease of use, lack of convenience, privacy and security | Cost |  |
|  |  | Lack of desire |  |
|  |  | Social implications |  |
|  |  | Technical literacy |  |
|  |  | Lack of tech support |  |
|  |  | Privacy and security |  |
| Moore AN, et al 2015 | computer literacy, computer anxiety, computer self-efficacy | Technical literacy |  |
|  |  | Computer anxiety |  |
|  |  | Lack of tech support |  |
| Currie M, et al 2015 | too impersonal | Social implications |  |
| Grant LA, et al 2015 | no perceived utility, cost, invasion of privacy | Lack of desire |  |
|  |  | Cost |  |
|  |  | Privacy and security |  |
| Brenes GA, et al 2015 | Not reported | Not reported |  |
|  |  |  |  |
|  |  |  |  |
|  |  |  |  |
| Corbett A, et al 2015 | Not reported | Not reported |  |
|  |  |  |  |
|  |  |  |  |
| Mavandadi S, et al 2015 | Not reported | Not reported |  |
|  |  |  |  |
|  |  |  |  |
| Egede LE, et al 2015 | Not reported | Not reported |  |
|  |  |  |  |
|  |  |  |  |
| Chang W, et al 2015 | Not reported | Not reported |  |
|  |  |  |  |
| Boulos M, et al 2015 | health literacy,  not useful information, confusing menus, hand tremors, small icons | Medical literacy |  |
|  |  | Lack of tech support |  |
|  |  | Mental acuity |  |
|  |  | Hand-eye coordination |  |
|  |  | Visual acuity |  |
| Dino M & deGuzman A 2015 | not useful, not effective, | Lack of desire |  |
|  |  | Lack of tech support |  |
|  |  |  |  |
| Czaja SJ, et al 2015 | no experience | Technical literacy |  |
|  |  |  |  |
|  |  |  |  |
| Choi NG, et al 2015 | no Internet access, not right for me | Ownership of technology |  |
|  |  | Lack of desire |  |
|  |  |  |  |
