# Supplementary material for: Utilization Barriers and Medical Outcomes Commensurate With the Use of Telehealth Among Older Adults: Systematic Review
Source: JMIR Med Inform. 2020 Aug 12;8(8):e20359. doi: 10.2196/20359 (PMC7450384; doi:10.2196/20359)
Supplement: Multimedia Appendix 4 [file medinform_v8i8e20359_app4.docx]

**Appendix D: Bias, Country of Origin, Statistics, and Quality assessment**

| Authors | Bias within study | Country of Origin | Statistics Used | JHNEDP Quality Assessment | |  |
| --- | --- | --- | --- | --- | --- | --- |
|  |  |  |  | Strength of Evidence | Quality of Evidence |  |
| Hamilton T, et al 2020 | Participants self-report  Participant functional level nor environmental factors not accounted for Recruitment concentrated on community centers & congregate housing which may not represent entire population | United States | Chi-square test; multivariate analysis | III | A |  |
|  |  |  |  |  |  |  |
| Theis S, et al 2019 | Participants came from the same country | Germany | Descriptive statistics; chi-square test; multiple linear regression; point-biseral correlation coefficient; simple linear regression | III | A |  |
|  |  |  |  |  |  |  |
|  |  |  |  |  |  |  |
|  |  |  |  |  |  |  |
| Wildenbos GA, et al 2019 | Participants came from the same country | Netherlands | Descriptives and kappa | III | B |  |
|  |  |  |  |  |  |  |
|  |  |  |  |  |  |  |
|  |  |  |  |  |  |  |
|  |  |  |  |  |  |  |
| Jakobsson E, et al 2019 | Sample was homogenous: suburban areas Participants came from the same region. | Sweden | Atlas.ti software analyzed transcripts | III | B |  |
|  |  |  |  |  |  |  |
|  |  |  |  |  |  |  |
|  |  |  |  |  |  |  |
|  |  |  |  |  |  |  |
|  |  |  |  |  |  |  |
|  |  |  |  |  |  |  |
| Karlsen C, et al 2019 | Norwegian speakers only | Norway | Descriptive statistics | III | B |  |
|  |  |  |  |  |  |  |
|  |  |  |  |  |  |  |
|  |  |  |  |  |  |  |
|  |  |  |  |  |  |  |
|  |  |  |  |  |  |  |
| Coley N, et al 2019 | 1- Selection bias because used different recruitment methods in each country which probably affected the between country comparisons | Finland, France, Netherlands | Kruskal-Willis tests; chi-squared test; Fisher exact test; structured content analysis | I | A |  |
| Giesbrecht & Miller 2019 | 1- Limited to English speaking participants | Canada | reliability for test-retest; intra-rater; inter-rater; one way & two way ANCOVA; pp analysis | I | A |  |
|  |  |  |  |  |  |  |
| Brodbeck J, et al 2019 | Limited to Bern, Switzerland and German speakers | Switzerland | Chi squared | I | A |  |
|  |  |  |  |  |  |  |
|  |  |  |  |  |  |  |
|  |  |  |  |  |  |  |
|  |  |  |  |  |  |  |
|  |  |  |  |  |  |  |
| Mosley C, et al 2019 | Study conducted in Seattle, WA only | USA | Linear regression | II | A |  |
| Jensen C, et al. 2019 | Convenience sample in one country only | Denmark | Language processing | II | A |  |
|  |  |  |  |  |  |  |
|  |  |  |  |  |  |  |
|  |  |  |  |  |  |  |
|  |  |  |  |  |  |  |
| Rasche P, et al 2018 | Convenience sample in one country only | Germany | logistics regression | II | A |  |
|  |  |  |  |  |  |  |
|  |  |  |  |  |  |  |
|  |  |  |  |  |  |  |
|  |  |  |  |  |  |  |
|  |  |  |  |  |  |  |
| Portz JD, et al 2018 | Small sample size | USA | chi-square and magnitude coding | III | B |  |
|  |  |  |  |  |  |  |
| Castro Sweet CM, et al 2018 | Observational design lends itself to selection bias as those who volunteered most likey had greater access to technology | USA | Paired t- test, and p test. | II | A |  |
|  |  |  |  |  |  |  |
|  |  |  |  |  |  |  |
| Joe J, et al 2018 | all participants were from a single metropolitan area and self-selected for participation, the generalizability of the study may not be strong | USA | None | III | B |  |
|  |  |  |  |  |  |  |
|  |  |  |  |  |  |  |
|  |  |  |  |  |  |  |
|  |  |  |  |  |  |  |
|  |  |  |  |  |  |  |
| Dham P, et al 2018 | All participants were from South Australia, which limits the external validity of the results | Australia | prospective post-consultation feedback, descriptive analysis, retrospective chart review, 5-point likert scale, Mann Whitney U-Test | III | A |  |
|  |  |  |  |  |  |  |
| Paige SR, et al 2018 | USA only, English speaking only | USA | survey | III | A |  |
|  |  |  |  |  |  |  |
| Cajita MI, 2018 | Secondary data analysis | USA | Descriptives | III | B |  |
|  |  |  |  |  |  |  |
|  |  |  |  |  |  |  |
|  |  |  |  |  |  |  |
|  |  |  |  |  |  |  |
|  |  |  |  |  |  |  |
| Harte R, 2018 | Participants recruited from same city | Ireland | satisfaction rating, time taken to complete tasks, cues required to complete tasks, and errors made during tasks. | III | B |  |
| Gordon NP & Hornbrook MC 2018 | Used patients in Kaiser Permanente system only | USA | Descriptives | III | B |  |
|  |  |  |  |  |  |  |
| Bao T, et al 2018 | Participants recruited from same city | USA | t-test | I | B |  |
|  |  |  |  |  |  |  |
|  |  |  |  |  |  |  |
| Egede LE, et al 2018 | Restricted to one VA centers in the USA | USA | Descriptives | I | A |  |
|  |  |  |  |  |  |  |
|  |  |  |  |  |  |  |
|  |  |  |  |  |  |  |
|  |  |  |  |  |  |  |
| Platts-Mills TF, 2018 | Only conducted at one facility | USA |  | I | A |  |
| Lopez-Villegas A, et al 2018 | Only conducted at one facility | Norway | Chi-squared and Fisher exact test | I | A |  |
| Dugas M, et al 2018 | Participants recruited from same city | USA | One-way ANOVA, linear mixed models | II | B |  |
|  |  |  |  |  |  |  |
| Nalder E, et al 2018 | Participants recruited from same city | Canada | Language processing | III | B |  |
|  |  |  |  |  |  |  |
|  |  |  |  |  |  |  |
|  |  |  |  |  |  |  |
| Buck H, et al 2017 | Small sample size | USA | Language processing | III | B |  |
|  |  |  |  |  |  |  |
| Ware P, et al 2017 | Small sample size | Canada | Language processing | IV | B |  |
|  |  |  |  |  |  |  |
|  |  |  |  |  |  |  |
|  |  |  |  |  |  |  |
|  |  |  |  |  |  |  |
|  |  |  |  |  |  |  |
| Chang CP, et al 2017 | Participants recruited from same city | Taiwan | Language processing | III | B |  |
|  |  |  |  |  |  |  |
| Cajita MI, et al 2017 | Convenience sample in one country only | USA | Linear regression | II | A |  |
|  |  |  |  |  |  |  |
|  |  |  |  |  |  |  |
|  |  |  |  |  |  |  |
|  |  |  |  |  |  |  |
| LaMonica HM, 2017 | Convenience sample in one city only | Australia | Descriptives and Fisher exact tests | III | A |  |
|  |  |  |  |  |  |  |
|  |  |  |  |  |  |  |
| Bahar-Fuchs A, et al 2017 | Convenience sample in one city only | Australia | Descriptives and Cohen's d | I | A |  |
|  |  |  |  |  |  |  |
|  |  |  |  |  |  |  |
|  |  |  |  |  |  |  |
| Nahm ES, et al 2017 | Convenience sample in one city and VA only | USA | Cohen's f and Cohen's d | I | A |  |
|  |  |  |  |  |  |  |
|  |  |  |  |  |  |  |
| Knaevelsrud C, et al 2017 | Participants recruited from same city | Germany | Chi-squared, Cohen's d | I | A |  |
|  |  |  |  |  |  |  |
|  |  |  |  |  |  |  |
|  |  |  |  |  |  |  |
| Reijnders JS, et al 2017 | Participants recruited from same city | Netherlands | Student t-test and chi squared | I | A |  |
|  |  |  |  |  |  |  |
|  |  |  |  |  |  |  |
| Mageroski A, et al 2016 | Participants recruited from same country | Australia | Person correlation | III | B |  |
|  |  |  |  |  |  |  |
| Hamblin K, et al 2016 | Participants recruited from same city | USA | Language processing | III | A |  |
|  |  |  |  |  |  |  |
|  |  |  |  |  |  |  |
|  |  |  |  |  |  |  |
| Wang J, et al 2016 | Small sample size.  Selection bias | USA | Descriptives | III | B |  |
| Gordon NP & Hornbrook MC 2016 | Participants all came from population of Kaiser Permanente hospital system | USA | Descriptives | II | A |  |
|  |  |  |  |  |  |  |
|  |  |  |  |  |  |  |
|  |  |  |  |  |  |  |
|  |  |  |  |  |  |  |
|  |  |  |  |  |  |  |
|  |  |  |  |  |  |  |
|  |  |  |  |  |  |  |
| Williams K, et al 2016 | small sample size | USA | Language processing | III | C |  |
|  |  |  |  |  |  |  |
|  |  |  |  |  |  |  |
|  |  |  |  |  |  |  |
|  |  |  |  |  |  |  |
| Evans J, et al 2016 | Participants recruited from same city | USA | Wilks' lambda with Bonferroni, Cohen's d | II | A |  |
|  |  |  |  |  |  |  |
|  |  |  |  |  |  |  |
|  |  |  |  |  |  |  |
| Muller AM, et al 2016 | Participants recruited from same city | Malaysia | student t-test, Cohen's d, Fisher's exact test | I | A |  |
|  |  |  |  |  |  |  |
|  |  |  |  |  |  |  |
|  |  |  |  |  |  |  |
|  |  |  |  |  |  |  |
|  |  |  |  |  |  |  |
| Quinn CC, et al 2016 | Participants recruited from same city | USA | linear mixed-effect model | I | A |  |
|  |  |  |  |  |  |  |
| Royackers A, et al 2016 | Participants recruited from same city | Canada | Language processing | III | B |  |
|  |  |  |  |  |  |  |
|  |  |  |  |  |  |  |
| Duh E, et al 2016 | Participants recruited from same city | Slovenia | Language processing | III | B |  |
|  |  |  |  |  |  |  |
|  |  |  |  |  |  |  |
|  |  |  |  |  |  |  |
|  |  |  |  |  |  |  |
| Depatie, A & Bigbee, JL 2015 | Convenience sample taken from one region of California | USA | descriptive statistics | II | A |  |
|  |  |  |  |  |  |  |
|  |  |  |  |  |  |  |
|  |  |  |  |  |  |  |
|  |  |  |  |  |  |  |
|  |  |  |  |  |  |  |
| Moore AN, et al 2015 | Participants recruited from same city | USA | correlation analysis with Bonferroni correction | III | B |  |
|  |  |  |  |  |  |  |
|  |  |  |  |  |  |  |
| Currie M, et al 2015 | Participants recruited from same city | Scotland | Language processing | II | B |  |
| Grant LA, et al 2015 | Participants recruited from same city | USA | descriptives and language processing | I | A |  |
|  |  |  |  |  |  |  |
|  |  |  |  |  |  |  |
| Brenes GA, et al 2015 | Participants recruited from same city | USA | Descriptives and student's t-test | I | A |  |
|  |  |  |  |  |  |  |
|  |  |  |  |  |  |  |
|  |  |  |  |  |  |  |
| Corbett A, et al 2015 | Participants recruited from same city | UK | Cohen's d | I | A |  |
|  |  |  |  |  |  |  |
|  |  |  |  |  |  |  |
| Mavandadi S, et al 2015 | Participants recruited from same city | USA | Descriptives, univariable analysis, Chi-squared, Cohen's d | I | A |  |
|  |  |  |  |  |  |  |
|  |  |  |  |  |  |  |
| Egede LE, et al 2015 | Participants recruited from same VA facility | USA | Descriptives, Fisher's exact test | I | A |  |
|  |  |  |  |  |  |  |
|  |  |  |  |  |  |  |
| Chang W, et al 2015 | Participants recruited from same city | Taiwan | Descriptives, student t-test | III | B |  |
|  |  |  |  |  |  |  |
| Boulos M, et al 2015 | Selection bias | EU | Language processing | III | B |  |
|  |  |  |  |  |  |  |
|  |  |  |  |  |  |  |
|  |  |  |  |  |  |  |
|  |  |  |  |  |  |  |
| Dino M & deGuzman A 2015 | Participants recruited from same region of country | Philipines | Partial least squares | III | A |  |
|  |  |  |  |  |  |  |
|  |  |  |  |  |  |  |
| Czaja SJ, et al 2015 | Participants recruited from same city | USA | mixed design repeated measures analysis of variance procedure | III | A |  |
|  |  |  |  |  |  |  |
|  |  |  |  |  |  |  |
| Choi NG, et al 2015 | Participants recruited from same city | USA | one-way ANOVA, χ2 tests, and t tests | III | B |  |
|  |  |  |  |  |  |  |
|  |  |  |  |  |  |  |
